# PKM2 induces mitophagy through the AMPK-mTOR pathway promoting CSFV proliferation

**DOI:** 10.1128/jvi.01751-23

**Published:** 2024-02-06

**Authors:** Xiaodi Liu, Quanhui Yan, Xueyi Liu, Wenkang Wei, Linke Zou, Feifan Zhao, Sen Zeng, Lin Yi, Hongxing Ding, Mingqiu Zhao, Jinding Chen, Shuangqi Fan

**Affiliations:** 1 College of Veterinary Medicine, South China Agricultural University, Guangzhou, Guang Dong, China; 2 State Key Laboratory of Swine and Poultry Breeding Industry, Agro-Biological Gene Research Center, Guangdong Academy of Agricultural Sciences, Guangzhou, China; University of Michigan Medical School, Ann Arbor, Michigan, USA

**Keywords:** cellular metabolism, classical swine fever virus, pyruvate kinase M2, mitophagy, AMPK-mTOR, viral infection

## Abstract

Viruses exploit the host cell’s energy metabolism system to support their replication. Mitochondria, known as the powerhouse of the cell, play a critical role in regulating cell survival and virus replication. Our prior research indicated that the classical swine fever virus (CSFV) alters mitochondrial dynamics and triggers glycolytic metabolic reprogramming. However, the role and mechanism of PKM2, a key regulatory enzyme of glycolytic metabolism, in CSFV replication remain unclear. In this study, we discovered that CSFV enhances PKM2 expression and utilizes PKM2 to inhibit pyruvate production. Using an affinity purification coupled mass spectrometry system, we successfully identified PKM as a novel interaction partner of the CSFV non-structural protein NS4A. Furthermore, we validated the interaction between PKM2 and both CSFV NS4A and NS5A through co-immunoprecipitation and confocal analysis. PKM2 was found to promote the expression of both NS4A and NS5A. Moreover, we observed that PKM2 induces mitophagy by activating the AMPK-mTOR signaling pathway, thereby facilitating CSFV proliferation. In summary, our data reveal a novel mechanism whereby PKM2, a metabolic enzyme, promotes CSFV proliferation by inducing mitophagy. These findings offer a new avenue for developing antiviral strategies.

**IMPORTANCE:**

Viruses rely on the host cell’s material-energy metabolic system for replication, inducing host metabolic disorders and subsequent immunosuppression—a major contributor to persistent viral infections. Classical swine fever virus (CSFV) is no exception. Classical swine fever is a severe acute infectious disease caused by CSFV, resulting in significant economic losses to the global pig industry. While the role of the metabolic enzyme PKM2 (pyruvate dehydrogenase) in the glycolytic pathway of tumor cells has been extensively studied, its involvement in viral infection remains relatively unknown. Our data unveil a new mechanism by which the metabolic enzyme PKM2 mediates CSFV infection, offering novel avenues for the development of antiviral strategies.

## INTRODUCTION

Classical swine fever is a severe acute infectious disease caused by the classical swine fever virus (CSFV), which has caused substantial economic losses to the global pig industry (1). CSFV, along with bovine viral diarrhea virus and hepatitis C virus (HCV), belongs to the Pestivirus genus in the *Flaviviridae* family (2). CSFV is an enveloped RNA virus with a single-stranded RNA genome of approximately 12.3 kb. A large open reading frame encodes a unique polyprotein, which undergoes translation and processing to produce four structural proteins (C, E^rns^, E1, and E2) and eight non-structural proteins (N^pro^, p7, NS2, NS3, NS4A, NS4B, NS5A, and NS5B) (3, 4). Among these, NS2, NS3, and NS5A are considered essential for viral replication (5). CSFV has developed mechanisms to evade host immune responses under immune selection pressure (6). Despite numerous studies investigating CSFV-host interactions (7), the pathogenesis and immune evasion strategies of CSFV remain unclear.

Viruses rely on the host’s material metabolism and energy supply to replicate, orchestrating an anabolic reprogramming of host cell metabolism. They depend on host cells to synthesize lipids, proteins, and nucleic acids for generating viral daughter precursors. Additionally, viruses require energy to drive processes such as replication, assembly, and release (8). During glucose metabolism, pyruvate is a critical intermediate in glycolysis. Numerous studies have shown that viruses can modify pyruvate levels in host cells upon infection. For instance, SARS-CoV-2 infection leads to elevated levels of host pyruvate, while white spot syndrome virus infection significantly increases lactate, a metabolite closely related to pyruvate metabolism, in blood cells. Additionally, adenovirus-mediated overexpression of MCT-1 resulted in heightened pyruvate oxidation and increased pyruvate-stimulated insulin release into isolated rat islets. Beyond its pro-tumor effects, pyruvate metabolism plays a crucial role in the regulation of both innate and adaptive immunity. This includes the production of IL-1β and IL-10 in macrophages, as well as the promotion of inflammatory responses (9, 10). Moreover, CSFV infection was observed to inhibit pyruvate release, underscoring the significance of pyruvate metabolism in the virus-host interaction. Besides their anabolic and catabolic functions, many metabolic intermediates can serve as crucial signaling molecules in immune regulation.

Pyruvate kinase (PK) plays a key role in catalyzing the formation of pyruvate and ATP from phosphoenolpyruvate and ADP (11). In the nucleus, PKM2 regulates cell proliferation (12) and binds directly to phosphorylated histone H3, thereby promoting gene transcription (13). Numerous studies have demonstrated that PKM2 is the predominant M isoform in most adult tissues (14, 15) and plays an essential role in the energy metabolism of both cancer cells and tumor cells (16). High expression of PKM2 is indeed associated with a poor prognosis in patients with various cancers and acute leukemia (17–20). In response to oxidative stress, PKM2 translocates to mitochondria and directly regulates apoptosis by phosphorylating Bcl2 (21). Additionally, PKM2 deficiency limits endothelial cell growth and triggers innate immune signaling in various cell types (22). Despite numerous studies highlighting the critical role of PKM2 in energy metabolism in cancer and tumor cells, the specific role of PKM2 in these metabolic alterations and their impact on viral replication have yet to be fully elucidated.

Mitochondria are central to cellular metabolism (23) and undergo continuous processes such as division, fusion, and phagocytosis to promote mitochondrial quality control (24). Abnormal mitochondrial dynamics have been associated with the pathogenesis of several genetic and neurological disorders, cardiac dysfunction, cancer, and metabolic diseases, including diabetes and obesity (25, 26). Mitochondrial dysfunction also accelerates the production of reactive oxygen species (ROS) and cytoplasmic release of cytochrome c, promoting programmed cell death and cardiomyocyte injury (27). Mitophagy, a process that maintains mitochondrial quality and quantity, involves delivering damaged mitochondria to lysosomes for degradation via double-membrane autophagosomes (28, 29). Various studies have found that viruses exploit mitophagy to facilitate their replication. For example, Newcastle disease virus (NDV), HCV, hepatitis B virus, and influenza virus have been shown to induce mitophagy, thereby suppressing natural immunity and creating an environment conducive to viral replication (30–32). Our previous study found that CSFV infection induces the formation of autophagosome-like vesicle membranes and hijacks the autophagic vesicle transport pathway for replication (33). Furthermore, CSFV infection promotes the mitochondrial translocation of PINK1 and Parkin, leading to ubiquitinated degradation of mitochondrial outer membrane proteins and inducing complete mitophagy (34). We also noted in our earlier work that CSFV infection influences pyruvate release (35). Considering that glycolysis is recognized as a significant energy source for mitochondrial metabolism in diseases such as cancer and diabetes (36), we hypothesize that PKM2 may impact mitochondrial biological function based on these results.

In this study, we investigated the mechanism by which the host cell protein PKM2 regulates mitophagy in response to CSFV infection. Our results demonstrated that CSFV modulated intracellular pyruvate content through PKM2, which interacts with and promotes the expression of both NS4A and NS5A. Additionally, we observed that PKM2 induced mitophagy via the AMPK-mTOR signaling pathway, thereby promoting CSFV proliferation. Interestingly, PKM2 facilitated the mitochondrial translocation of AMPK during this process. These findings not only shed light on the role of PKM2 in CSFV proliferation but also provide new insights into the potential for controlling the disease.

## RESULTS

### CSFV infection promoted the expression of PKM2 both *in vitro* and *in vivo*

**Fig 1:**
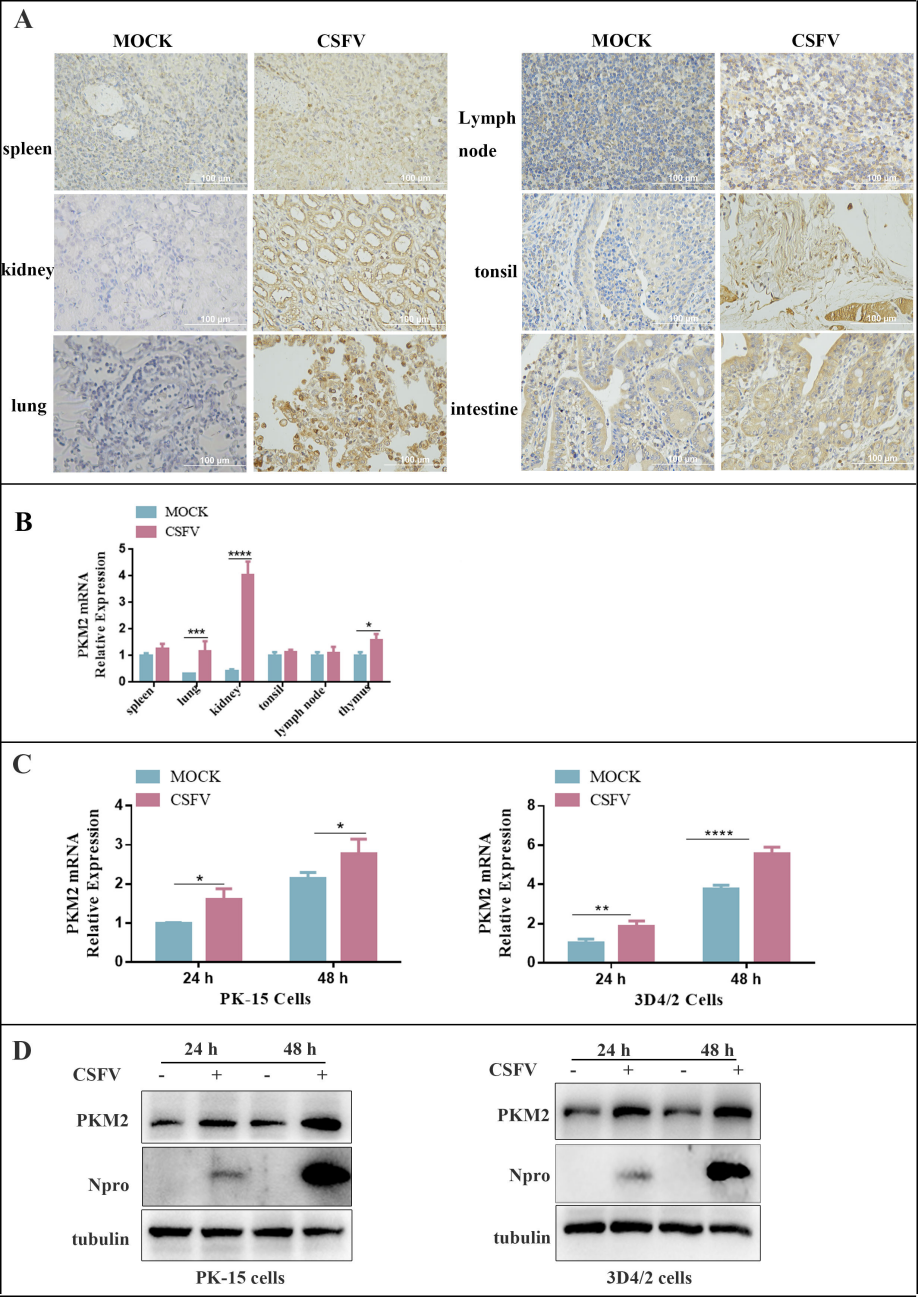
CSFV infection upregulated PKM2 expression. (**A**) Immunohistochemical analysis of PKM2 expression in normal and CSFV-infected tissues. (**B**) RT-qPCR analysis of PKM2 gene expression in normal and CSFV-infected tissues. Error bars indicate the mean (±SD) of three independent experiments. * *P* < 0.05; ***P < 0.001; ****P < 0.0001, and ns, *P* > 0.05 (one-way ANOVA). (**C**) RT-qPCR analysis of PKM2 gene transcription in CSFV-infected PK-15 and 3D4/2 cells. Error bars indicate the mean (±SD) of three independent experiments. *n* = 3. *P < 0.05; **P < 0.01; ***P < 0.001; and ns, *P* > 0.05 (one-way ANOVA). (**D**) Western blot analysis of PKM2 protein expression in CSFV-infected PK-15 and 3D4/2 cells. The level of protein was quantified using Image-Pro Plus 6.0 software, and the ratios were calculated relative to the tubulin control. Error bars indicate the mean (±SD) of three independent experiments. ****P < 0.0001 (one-way ANOVA).

Our previous metabolomic studies revealed that CSFV infection induces a metabolic reprogramming of the pyruvate pathway in PK-15 and 3D4/2 cells (37). To further investigate the effect of CSFV infection on PKM2 protein expression profiles in host cells *in vivo*, we conducted an animal experiment using 30-day-old piglets without specific pathogens. The piglets were infected with CSFV, while normal piglets served as the control group. After the onset of disease in piglets, we measured PKM2 content through immunohistochemistry and quantitative real-time RT-PCR (RT-qPCR) in tissues at the lesion site, including immune tissues such as spleen, tonsils, and lymph nodes, versus non-immune tissues, such as the lungs and kidneys. Our results revealed a significant increase in PKM2 levels in several tissues of infected piglets compared to normal piglets (Fig. 1A and B). The PK-15 cell line, initially isolated and cultured from porcine kidney tissues, is sensitive to CSFV and commonly used as a model cell line for the study of CSFV infection (38). 3D4/2, a cell line derived from porcine macrophage, was chosen as the target cells of CSFV infection (39). Given the noncytopathogenic nature of CSFV (40), we used the same multiplicity of infections (MOIs) and time of infection in PK-15 and 3D4/2.

*In vitro*, PK-15 and 3D4/2 cells were infected with CSFV at an MOI of 1.0, while non-infected cells served as controls. The replication cycle of CSFV was approximately 10 h, with peak titers observed 48 h post-infection in PK-15 and 3D4/2 cells (34). RNA and protein were collected from the cells at 24 and 48 h post-infection. Alterations in PKM2 levels were detected by RT-qPCR and western blot. The results showed a significant elevation in mRNA levels (Fig. 1C) and protein (Fig. 1D) of PKM2 in CSFV-infected PK-15 and 3D4/2 cells, indicating that CSFV infection *in vitro* promotes the expression of PKM2. These findings collectively suggest that CSFV infection alters the expression of PKM2 in piglet tissues and *in vitro* in PK-15 and 3D4/2 cells.

### CSFV exploited PKM2 to modulate pyruvate metabolism

**Fig 2:**
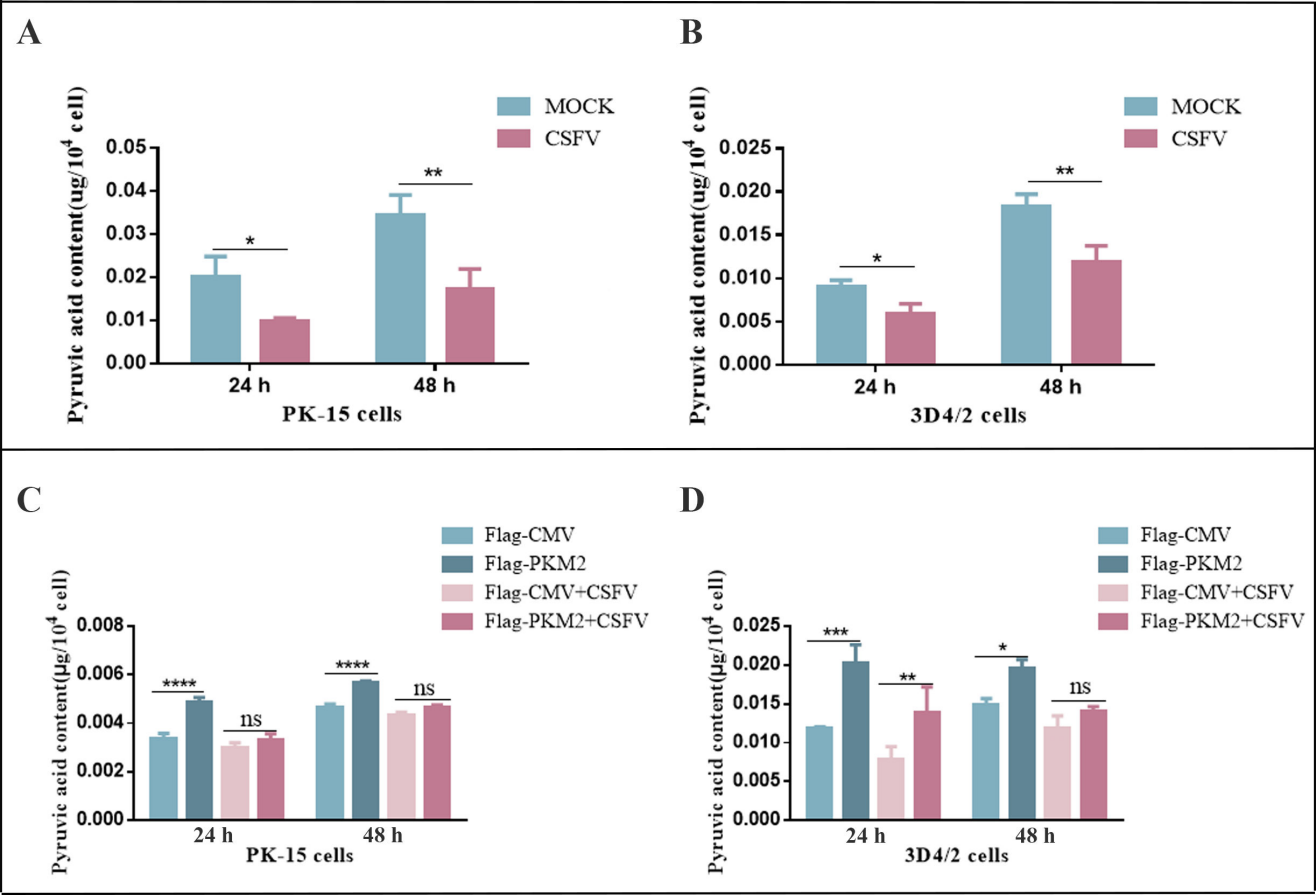
CSFV affects pyruvate metabolism in PK-15 and 34D/2 cells through PKM2. (**A and B**) PK-15 (**A**) and 3D4/2 cells (**B**) were infected with CSFV at an MOI of 1.0 and pyruvate content was measured by spectrophotometric assays in cultured cells at 24 and 48 h. (**C**) PK-15 cells were transfected with PKM2 and then infected with CSFV at an MOI of 1.0. The content of pyruvate in cultured cells was measured spectrophotometrically at 24 and 48 h. (**D**) 3D4/2 cells were transfected with PKM2 and then infected with CSFV at MOI of 1.0. The content of pyruvate in cultured cells was measured spectrophotometrically at 24 and 48 h. All measurements were made in triplicates. Error bars indicate the mean (±SD) of three independent experiments. *P < 0.05; **P < 0.01; and ***P < 0.001 (one-way ANOVA).

To investigate the impact of CSFV infection on pyruvate metabolism, we infected PK-15 and 3D4/2 cells with CSFV at an MOI of 1.0 and measured pyruvate levels in the cells at 24 and 48 h. The results demonstrated a reduction in pyruvate levels in CSFV-infected cells compared to uninfected cells (Fig. 2A and B). We further examined whether the effect of CSFV infection on pyruvate level was related to the regulatory enzyme PKM2. To investigate this, PK-15 and 3D4/2 cells were transfected with p3×Flag-PKM2 and infected with CSFV for 24 and 48 h. Overexpression of PKM2 increased pyruvate content in PK-15 and 3D4/2 cells compared to the control group, while CSFV infection attenuated the promotion effect of PKM2 on pyruvate (Fig. 2C and D). These results suggested that CSFV exploited PKM2 to influence pyruvate metabolism in PK-15 and 3D4/2 cells.

### PKM2 interacted with NS4A and NS5A

**Fig 3:**
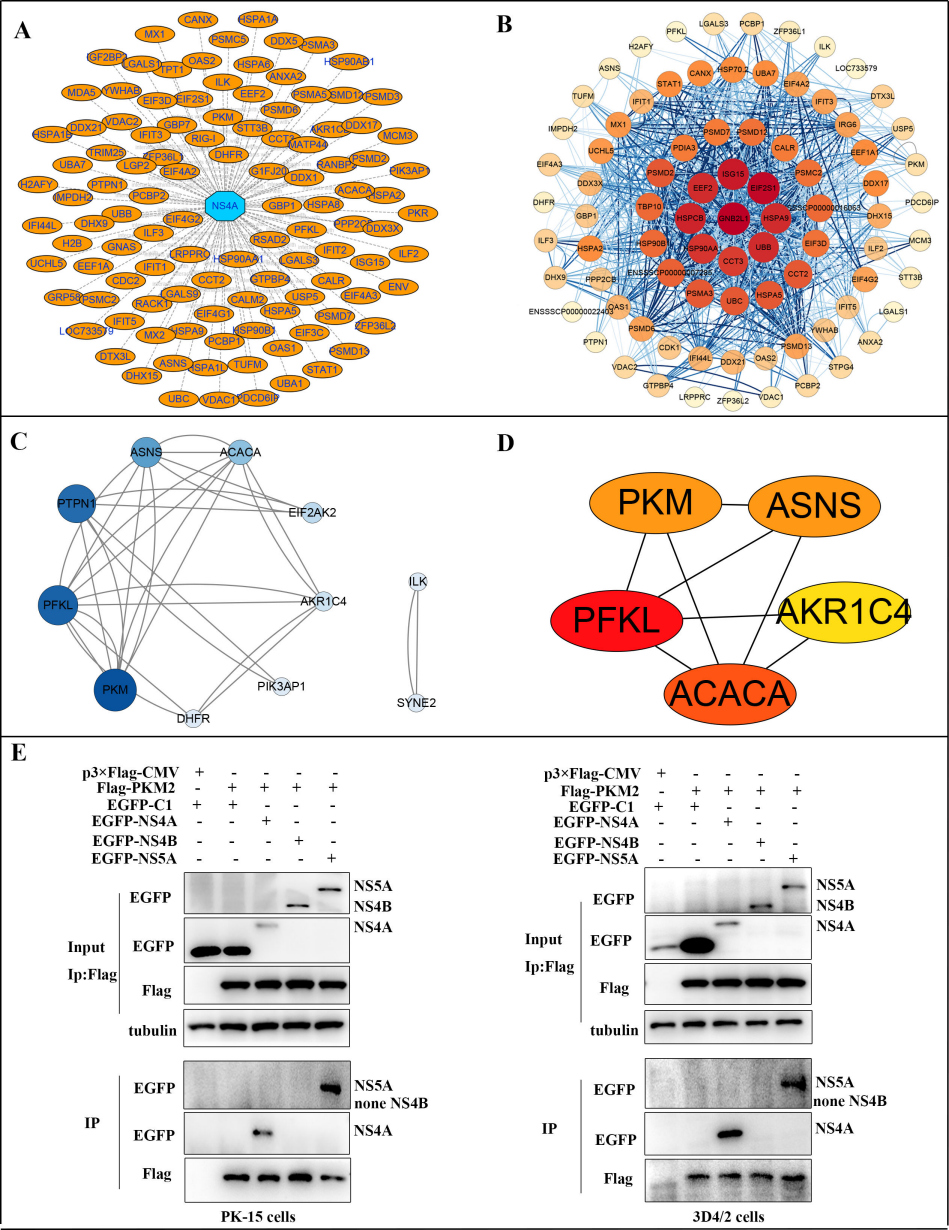
Validation of the interaction of PKM2 with NS4A and NS5A by mass spectrometry and immunoprecipitation. (**A**) Mass spectrometry screening of host-protein networks interacting with CSFV NS4A. (**B**) Analysis of the CSFV NS4A protein-host protein interaction network. (**C**) Statistical analysis of host proteins related to metabolism based on MCC using the cytoHubba plugin. (**D**) Statistical analysis of host proteins related to metabolism by setting continuous gradients based on degree values. (**E**) Co-immunoprecipitation analysis of 3×Flag-tagged PKM2 and GFP-tagged NS4A and NS5A by the anti-flag monoclonal antibody (mAb) or by the anti-GFP mAb. PK-15 and 3D4/2 cells were co-transfected with the indicated plasmids (+) or empty vectors (−) for 24 h. The transfected cells were lysed and incubated with a mouse anti-Flag mAb or anti-GFP mAb, followed by incubation with the protein G-agarose for 6 h at 4°C. The immunoprecipitate was analyzed by western blot using the anti-Flag and anti-GFP.

In our previous study, we identified PKM as one of the specific binding partners of the CSFV non-structural protein 4A (NS4A) through mass spectrometry screening, a protein critical to the replication cycle of CSFV (Table S1). We visualized the interactions between NS4A and the 112 host proteins using Cytoscape 3.7.2 software, as depicted in Fig. 3A. The blue octagon represents CSFV NS4A protein, while the orange oval represents the host protein screened by mass spectrometry to interact with NS4A. The interactions between these 83 host proteins were predicted using the STRING database (version 11.5), resulting in 941 pairs of protein-protein interactions. We generated the network diagram of these interactions using Cytoscape software (Fig. 3B). To delve into the metabolic relevance, we identified 11 of these host proteins as metabolism-related proteins. Utilizing Cytoscape software, we assessed all indicators of the interaction networks involving these metabolism-related host proteins. The top five proteins were calculated based on the Maximal Clique Centrality (MCC) using the cytoHubba plug-in, with PKM2 ranking third (Fig. 3C). We established a continuous gradient based on the degree value, revealing that the PKM2 degree value was the highest in this reciprocal network (Fig. 3D), indicating that PKM2 is a crucial protein associated with metabolism in this network. Studies have highlighted the significance of CSFV NS4B and NS5A proteins in viral replication and pathogenicity, with NS5A potentially functioning as a component of NS4B-related complexes (41). To further confirm their interaction, co-immunoprecipitation (co-IP) experiments were performed with PK-15 and 3D4/2 cells transiently co-expressing 3× Flag-tagged PKM2 and GFP-tagged NS4A/NS4B/NS5A. After co-IP with anti-GFP and anti-Flag, respectively, 3×Flag-PKM2 was found to form a complex with GFP-NS4A and GFP-NS5A but not with GFP-C1 and GFP-NS4B (Fig. 3E).

### PKM2 colocalized with CSFV NS4A and NS5A and promoted the expression of NS4A and NS5A

**Fig 4:**
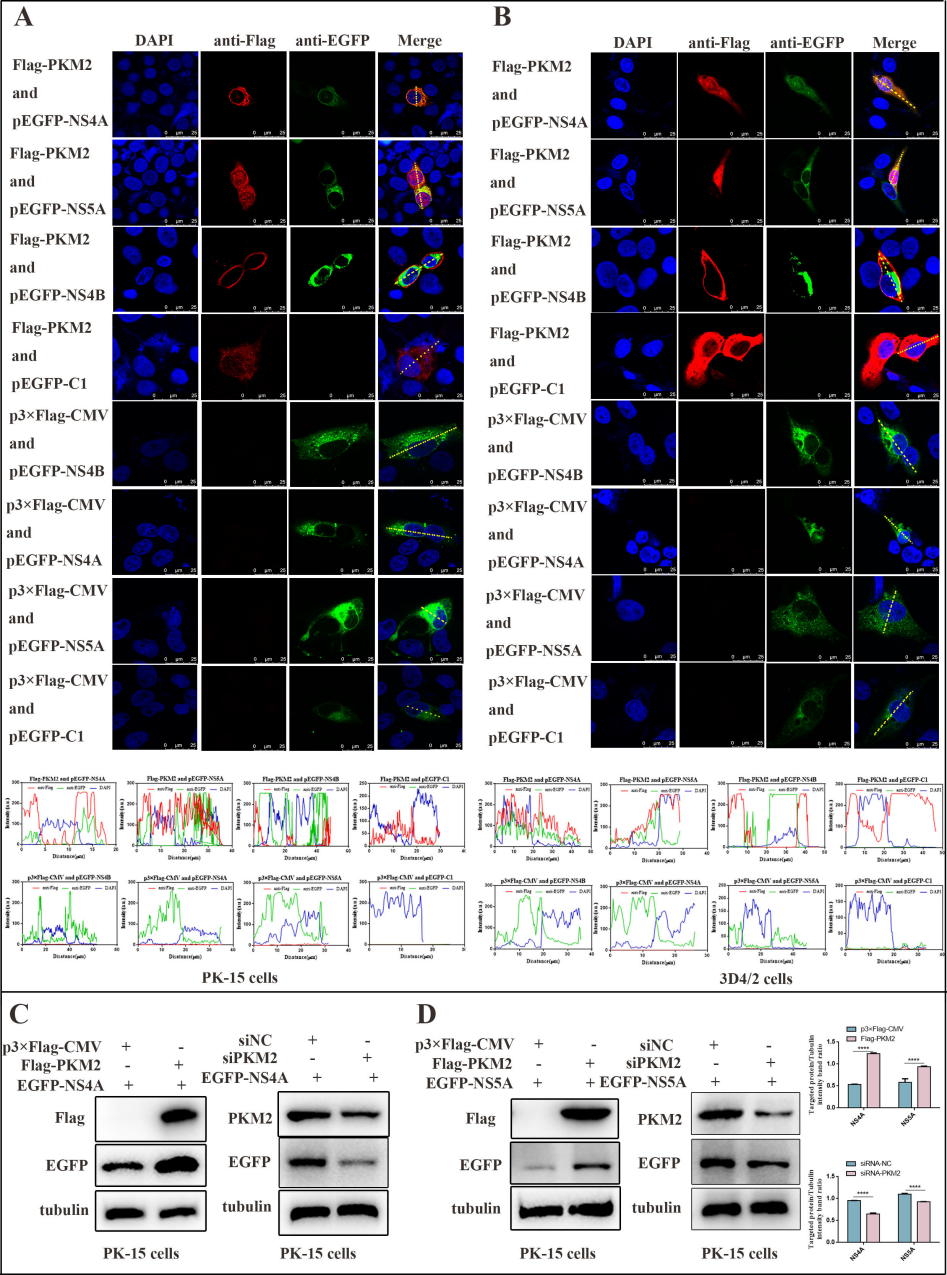
PKM2 colocalized with CSFV NS4A and NS5A and promoted the expression of NS4A and NS5A (**A and B**). PK-15 (**A**) and 3D4/2 (**B**) cells were co-transfected with 3× Flag-tagged PKM2 and GFP-tagged NS4A/NS5A. Cells were fixed at 24 h post-transfection and subjected to indirect immunofluorescence assay to detect GFP-NS4A/NS5A (green) and 3× Flag-PKM2 (red) with mouse anti-Flag and rabbit anti-GFP antibodies. The merged image indicates the nucleus by 4′,6-diamidino-2-phenylindole (DAPI) (blue) staining. (**C**) Western blot detection of NS4A protein expression levels in PK-15 cells overexpressing or silencing PKM2. (**D**) Western blot detection of NS5A protein expression levels in PK-15 cells overexpressing or silencing PKM2.

Following the identification of the direct interaction between PKM2 and CSFV NS4A or NS5A, we delved into the intracellular localization of PKM2 alongside NS4A/NS4B/NS5A proteins. PK-15 and 3D4/2 cells co-expressing NS4A /NS4B /NS5A and PKM2 were analyzed by confocal laser microscopy. Confocal images revealed PKM2 expression in the cytoplasm with perinuclear aggregation. Notably, NS4A and NS5A precisely co-localized with PKM2 in the same cytoplasmic distribution, while PKM2 did not exhibit co-localization with NS4B. Various control groups were established for comparison (Fig. 4A and B).

These results provide additional support for the interaction between PKM2 and NS4A /NS5A proteins. To validate the impact of PKM2 on the expression of NS4A and NS5A, we conducted co-transfections using p3×Flag-PKM2 along with the EGFP-NS4A or EGFP-NS5A plasmid in PK-15 cells. It was found that overexpression of PKM2 promoted the expression of NS4A and NS5A in PK-15 cells while silencing PKM2 inhibited the expression of NS4A and NS5A in PK-15 cells (Fig. 4C and D).

### PKM2 promoted the proliferation of CSFV

**Fig 5:**
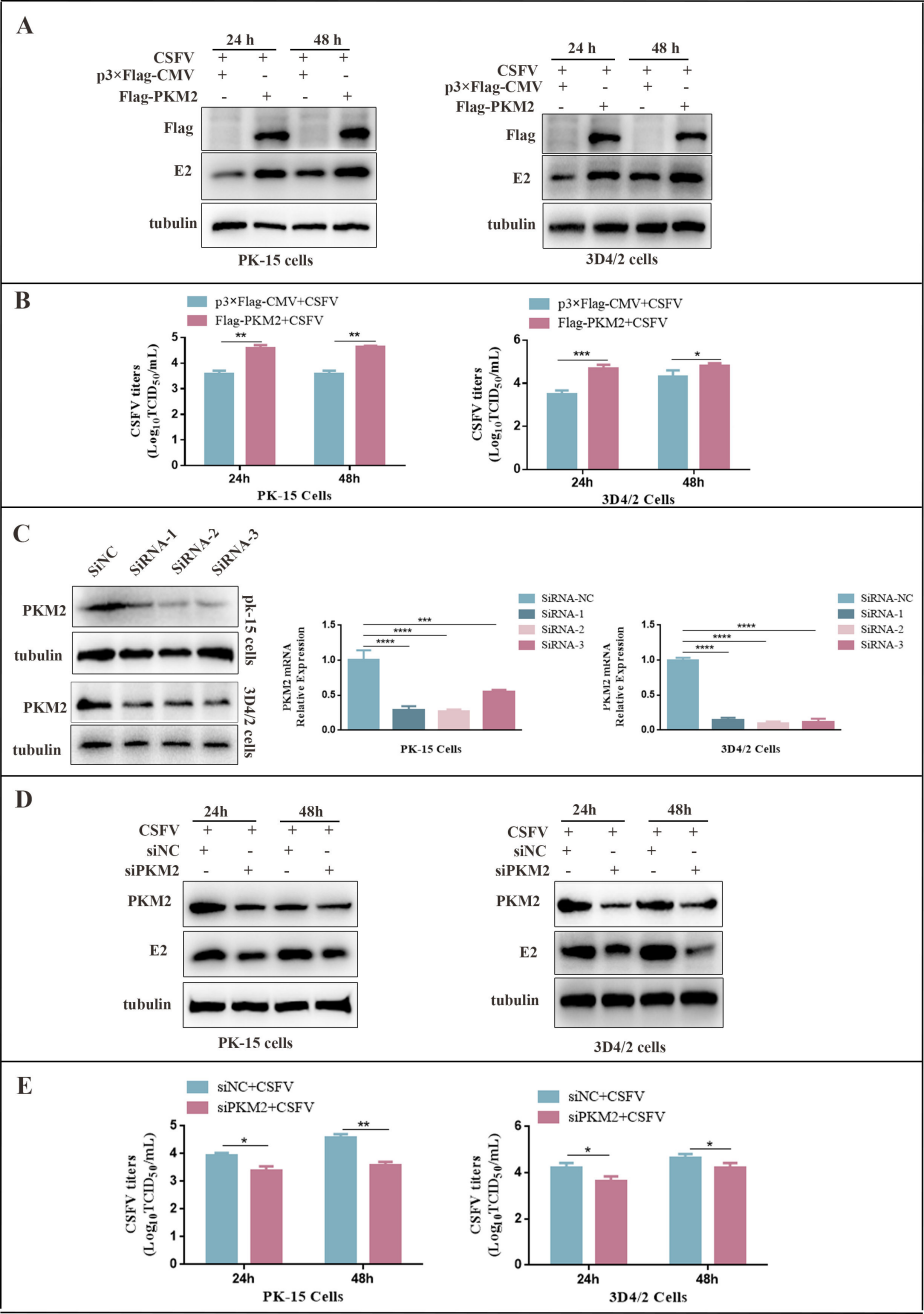
PKM2 positively regulated the proliferation of CSFV in PK-15 and 3D4/2 cells (**A and D**). PK-15 and 3D4/2 cells were transduced with p3×Flag-PKM2 or p3×Flag-CMV (**A**) (siNC or siPKM2) (**D**), followed by infection with CSFV at an MOI of 1.0 or mock infected. Cell samples were analyzed by western blot with antibodies against PKM2, CSFV E2, and tubulin (loading control). (**B and E**) CSFV virus titers in the supernatant were determined as 50% tissue culture infective doses (TCID50)/mL as described in Materials and Methods. Error bars indicate the mean (±SD) of three independent experiments. *P < 0.05; **P < 0.01; and ***P < 0.001 (one-way ANOVA). (**C**) siRNA knockdown of PKM2 in PK-15 and 3D4/2 cells transfected with siNC or PKM2 siRNA-1/-2/-3. The expression of PKM2 was assessed by western blot and RT-qPCR at 24 h. Error bars indicate the mean (±SD) of three independent experiments. ***P < 0.001 and ****P < 0.0001 (one-way ANOVA).

To assess the potential effect of PKM2 on CSFV proliferation, PK-15 and 3D4/2 cells were transfected with p3×Flag-PKM2 and p3×Flag-CMV, followed by infection with CSFV (MOI = 1.0) for 24 and 48 h. Subsequently, the expression of E2 protein and viral titers were examined by western blot and immunofluorescence assay (IFA), respectively. The results showed a significant increase in E2 protein expression (Fig. 5A) and elevated viral titers (Fig. 5B) after the transfection of p3×Flag-PKM2 in both PK-15 and 3D4/2 cells. These results demonstrate the promoting effect of PKM2 on CSFV proliferation. To further investigate the promoting effect of PKM2 on CSFV proliferation, three siRNAs were employed to knock down the expression of PKM2 in PK-15 and 3D4/2 cells. The efficacy of siRNAs in silencing the PKM2 protein expression and gene expression was confirmed by western blot and RT-qPCR, respectively (Fig. 5C). Subsequently, the cells were infected with CSFV (MOI = 1.0) for 24 and 48 h following PKM2 silencing. The E2 protein expression and viral titers of CSFV were analyzed by western blot and IFA, respectively. The knockdown of PKM2 expression, resulting in decreased E2 protein expression (Fig. 5D) and CSFV titers (Fig. 5E), was notably observed in PK-15 and 3D4/2 cells. These data provide additional support for the positive role of PKM2 in CSFV proliferation.

### PKM2 affected the proliferation of CSFV via pyruvate

Given that PKM2 catalyzes the conversion of phosphoenolpyruvate to pyruvate and influences CSFV replication, we aimed to explore the correlation between PKM2’s effect on CSFV replication and the production of pyruvate. To investigate this, we examined whether pyruvate affects the replication of CSFV. Since the cell culture medium we used in the above study contained some pyruvate, we replaced the cell culture medium with pyruvate-free Dulbecco’s modified eagle medium (DMEM) medium. Subsequently, we supplemented the pyruvate-free medium with pyruvate solutions to achieve the final concentrations of 0, 2, 5, 8, and 10 mmol/L. After 48 h, cells were collected and analyzed for the expression of CSFV N^pro^ protein and NS5B gene levels through western blot and RT-qPCR, respectively.

**Fig 6:**
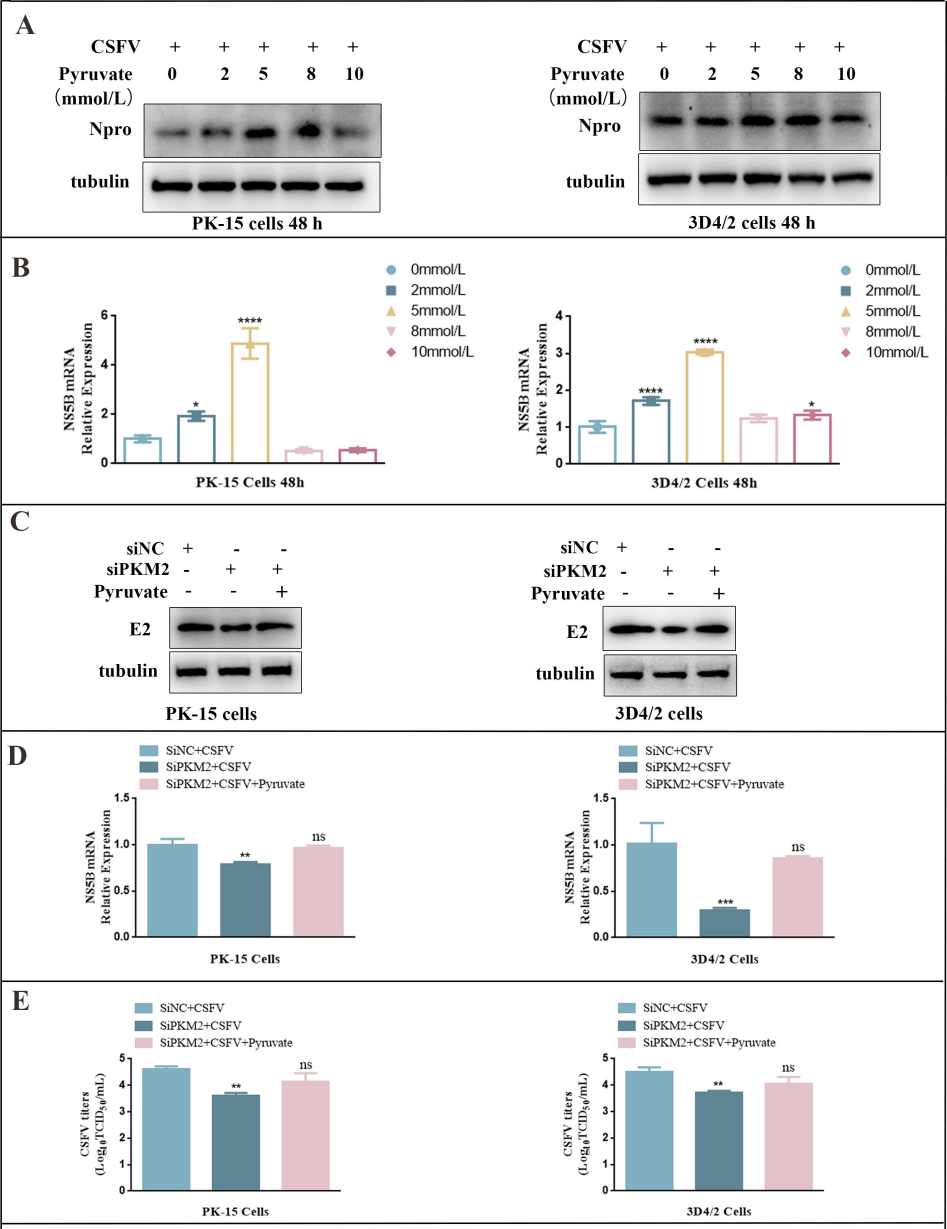
Rescue effect of pyruvate on silencing PKM2-induced inhibition of CSFV replication. (**A**) PK-15 and 3D4/2 cells were cultured in a pyruvate-free medium for a while after the addition of pyruvate solution, respectively, followed by infection with CSFV at an MOI of 1.0, and CSFV N^pro^ protein expression was detected after 48 h. (**B**) The relative expression level of NS5B gene in PK-15 and 3D4/2 cells was analyzed by RT-qPCR. Cells were treated as in panel **A**. (**C**) PK-15 and 3D4/2 cells were treated with pyruvate (5 mM) after being transfected with siPKM2 and then infected with CSFV at an MOI of 1.0. At 48 hpi, cell samples were analyzed by western blot with antibodies against CSFV E2 and tubulin (loading control). (**D**) PK-15 and 3D4/2 cells were treated as in panel **C**. NS5B gene levels in PK-15 and 3D4/2 cells were assessed using RT-qPCR. Error bars indicate the mean (±SD) of three independent experiments. ns, *P* > 0.05; **P < 0.01; and ***P < 0.001 (one-way ANOVA). (**E**) PK-15 and 3D4/2 cells were treated as in panel **C**. CSFV virus titers in the supernatant were determined as 50% tissue culture infective doses (TCID50)/mL as described in Materials and Methods. Error bars indicate the mean (±SD) of three independent experiments. ns, *P* > 0.05 and **P < 0.01 (one-way ANOVA).

Our findings revealed that pyruvate promoted CSFV replication via PKM2 in a dose-dependent manner, with the most significant promotion observed at 5 mmol/L pyruvate. Beyond 5 mmol/L, CSFV replication exhibited a reduction (Fig. 6A and B). Therefore, we selected 5 mmol/L as the final treatment concentration of pyruvate for subsequent study.

We then investigated whether 5 mM pyruvate could rescue the inhibition of viral replication caused by PKM2 silencing. We set up siPKM2 alone and pyruvate co-treatment with siPKM2 as well as a negative control group to detect the changes in the CSFV NS5B gene and viral titer under various treatments. The results demonstrated that the knockdown of PKM2 inhibited CSFV E2 protein expression level, NS5B gene expression, and viral titer compared with the control group, while 5 mM pyruvate had a significant back-compensation effect on the reduction of E2 protein expression (Fig. 6C), NS5B gene expression (Fig. 6D), and viral titer (Fig. 6E) caused by the knockdown of PKM2. It showed that 5 mM pyruvate could attenuate the inhibitory effect of knockdown of PKM2 on CSFV proliferation.

### PKM2 altered mitochondrial biology

**Fig 7:**
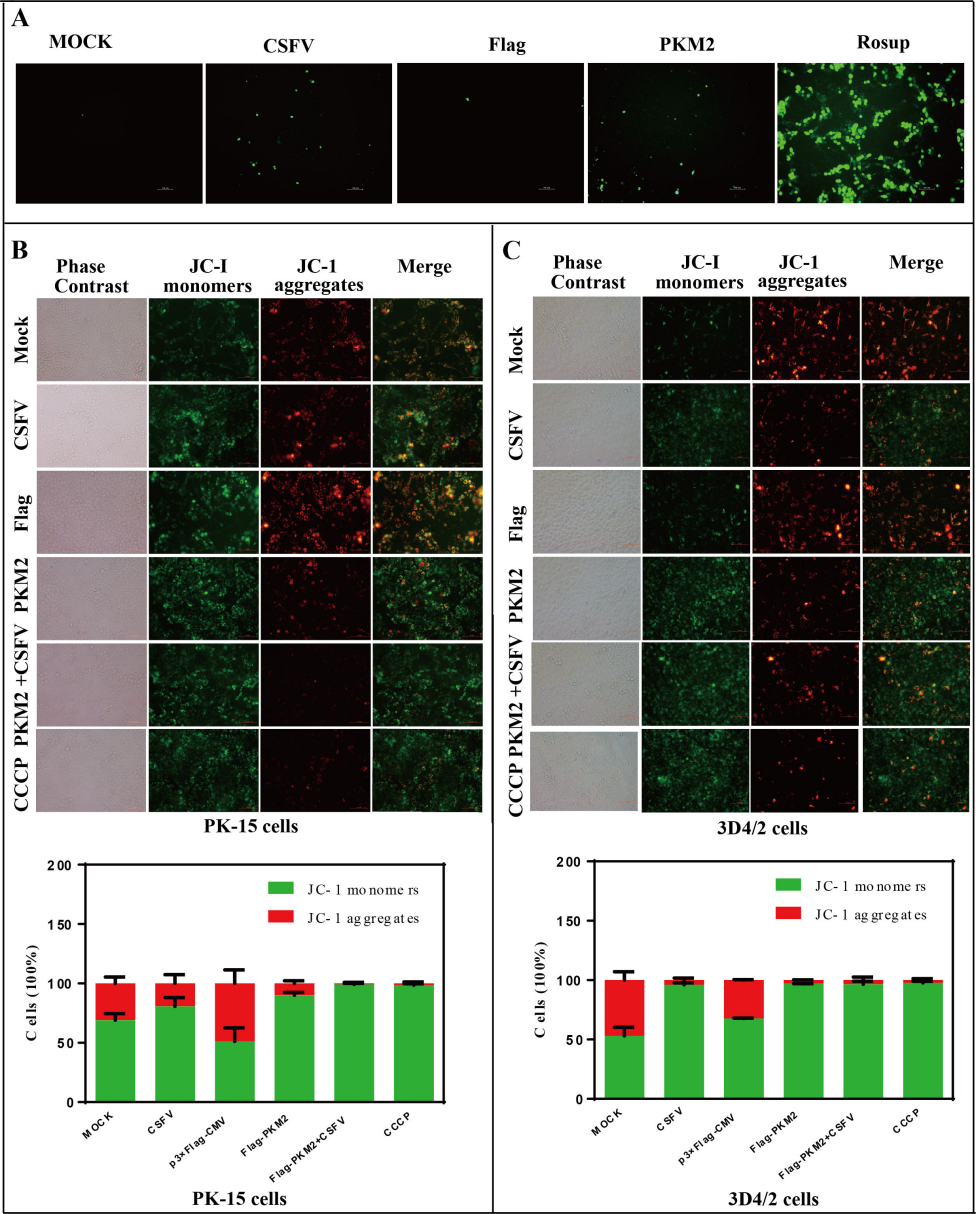
PKM2 increased ROS in cells and disrupted mitochondrial function. (**A**) PKM2 overexpression and CSFV infection increased ROS in PK-15 cells. Rosup as a positive control. (**B and C**) The Mitochondrial Membrane Potential Assay Kit (JC-1) detected mitochondrial membrane potential in PK-15 (**B**) and 3D4/2 cells (**C**). In normal mitochondria, JC-1 is present as a polymer with bright red fluorescence and very weak green fluorescence in the cell. When the mitochondrial membrane potential is reduced by treatment with carbonyl cyanide m-chlorobenzyl hydrazone (CCCP), JC-1 cannot be present as a polymer in the mitochondrial matrix, and the intensity of red fluorescence in the mitochondria is significantly reduced. In contrast, green fluorescence in the cytoplasm is enhanced considerably. Image-Pro Plus 6.0 software was used to calculate the mean fluorescence intensity of the line profile of the merged image (three times).

Mitochondria, ubiquitous in most mammalian cells, serve as the primary site for the production of reactive oxygen species, playing a crucial role in various pathological conditions and mediating redox signaling within the cell (42, 43). The dysfunction of mitochondria can lead to the loss of membrane potential (ΔΨm), resulting in the release of ROS, which in turn causes oxidative damage to cellular components, including proteins, lipids, and DNA (44). To investigate the impact of CSFV and PKM2 on cellular ROS levels, we measured the generation of ROS in PK-15 cells after infection with CSFV and transfection with p3×Flag-PKM2. Our findings indicated that both CSFV infection and PKM2 overexpression promoted the production of cellular ROS (Fig. 7A).

Mitochondrial damage often accompanies a decrease in mitochondrial membrane potential. To further elucidate the effects of PKM2 on mitochondria, we examined the changes in mitochondrial membrane potential levels in PK-15 cells and 3D4/2 cells. We employed JC-1, a versatile fluorescent probe utilized for assessing mitochondrial membrane potential (∆Ψm). In healthy cells, where ∆Ψm remains high, JC-1 forms multimers within the mitochondrial matrix, yielding a red fluorescence signal. Conversely, when ∆Ψm diminishes, JC-1 assumes a monomeric state within the mitochondrial matrix, resulting in a green fluorescence emission. The results indicated a notable reduction in the proportion of red fluorescence and a significant increase in green fluorescence in both CSFV-infected and PKM2-overexpressed groups compared to the control group. Moreover, the proportion of red fluorescence was lower in the group co-expressing PKM2 and infected with CSFV (Fig. 7B and C). These observations parallel those seen with carbonyl cyanide m-chlorobenzyl hydrazone (CCCP) treatment and suggest potential mitochondrial abnormalities.

### Overexpression of PKM2 induced mitochondrial fission and mitophagy

**Fig 8:**
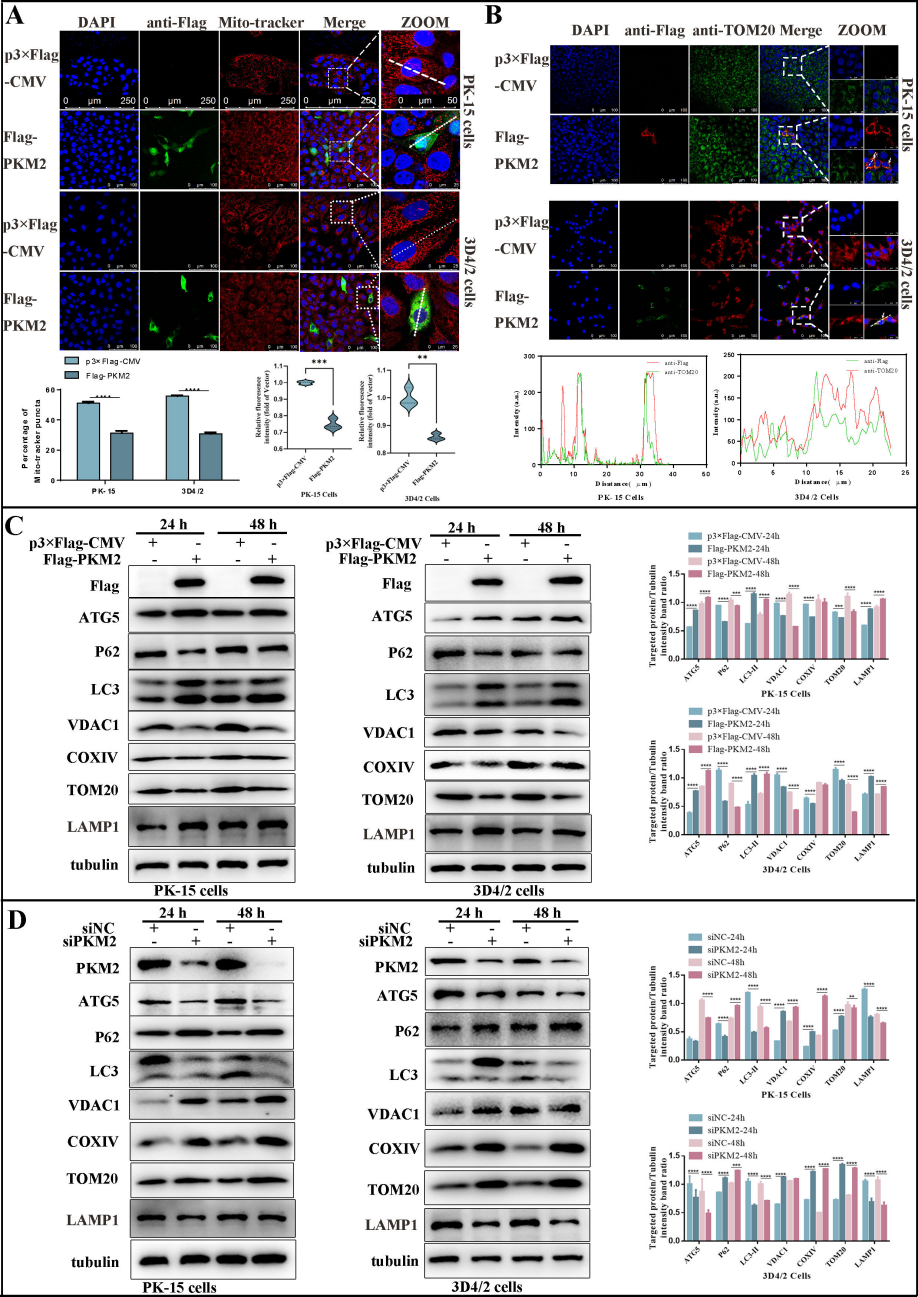
PKM2 induced mitochondrial fission and mitophagy. (**A**) Confocal microscopy images showing mitochondrial fragmentation in PKM2-overexpressed cells. PK-15 and 3D4/2 cells were transfected with P3×Flag-CMV or Flag-PKM2 for 24 h. Cells have stained the mitochondria with MitoTracker (red) and the cell nuclei with 4′,6-diamidino-2-phenylindole (DAPI) (blue). In the zoomed images, typical tubular mitochondria in control cells and fragmented mitochondria in PKM2-overexpressed cells are shown. The bar graph represents the average number of mitochondria (red dots) in each cell. Results represent the mean of at least three independent experiments. **P < 0.01; ***P < 0.001, and ****P < 0.0001. (**B**) Confocal microscope image showing co-localization of PKM2 with TOM20. Cells were prepared as in panel **A**. At 24 h, cells were immunostained with the TOM20 antibody (red) and 4′,6-diamidino-2-phenylindole (DAPI) (blue). Image-Pro Plus6.0 software was used to calculate the mean fluorescence intensity of the line profile of the merged image (three times). **P < 0.01 and ***P < 0.001 (one-way ANOVA). (**C and D**) Western blot detected the relative expression of the autophagy-associated proteins ATG5, LC3, and p62 in cells with PKM2 overexpression (**C**) and inhibition (**D**). The marker proteins TOM20, VDACI, COXIV, LAMP1, and tubulin (loading control) of mitophagy were also detected by western blot. The level of protein and fluorescence intensity were quantified using Image-Pro Plus 6.0 software. Error bars indicate the mean (±SD) of three independent experiments. **P < 0.01; ***P < 0.001, and ****P < 0.0001 (one-way ANOVA).

**Fig 9:**
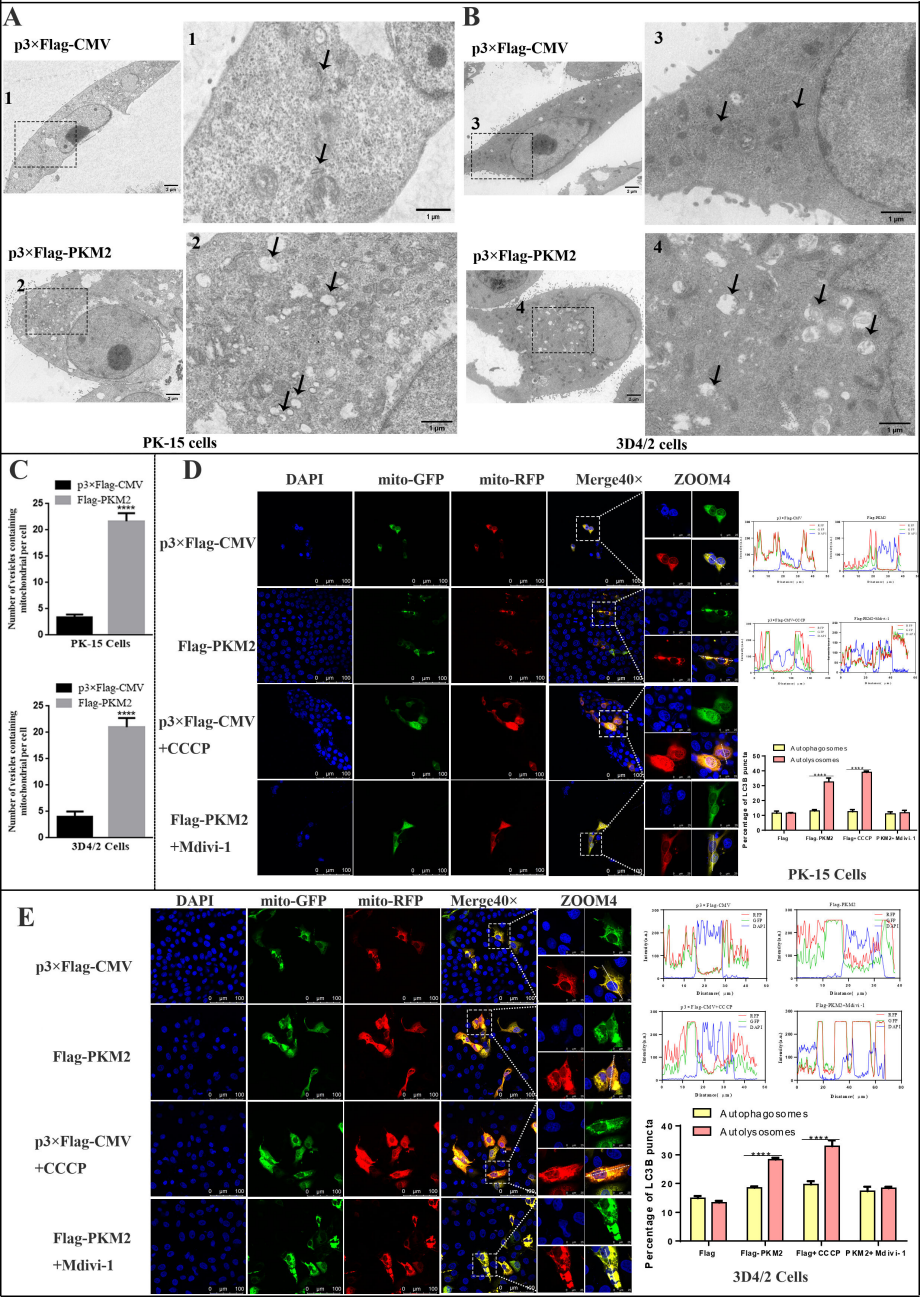
PKM2 induced complete mitophagy. (**A and B**) Overexpression of PKM2 increased mitochondrial autophagic vesicle formation in PK-15 (**A**) and 3D4/2 cells (**B**). TEM images revealed the mitochondrial ultrastructure in PKM2-overexpressed cells. PK-15 and 3D4/2 cells were mock handled or PKM2 overexpressed for 24 h and analyzed by TEM. Typical elongated tubular mitochondria in mock cells and fragmented elliptic mitochondria engulfed with membrane-like vesicles in PKM2-overexpressed cells were observed in the zoomed images. Scale bar: 2 µm. (**C**) Quantification of the mitophagosome-like vesicles per cell image (mean ± SD; *n* ≥ 5 cells; ****P < 0.0001) (one-way ANOVA). (**D and E**) PK-15 (**D**) and 3D4/2 cells (**E**) transiently expressing Mito-mRFP-EGFP were transfected with P3×Flag-CMV or Flag-PKM2 for 24 h. In the zoomed images, fluorescence signals indicated the expression of mRFP and GFP protein targeting mitochondria: yellow color, no mitophagy; red color, mitophagy. Image-Pro Plus 6.0 software was used to measure the fluorescence intensity quantitatively. Bar graphs represent the mean number of autophagosomes (puncta with both red and green colors, i.e., puncta with yellow color in merged images) and autolysosomes (puncta with only red but not green color, i.e., puncta with red color in merged images) per cell. Error bars indicate the mean (±SD) of three independent experiments. ns, *P* > 0.05 and ****P < 0.0001 (two-way ANOVA).

Building upon our prior studies that highlighted the pivotal role of mitophagy in CSFV infection, we delved into the question of whether PKM2 modulates mitochondria to influence CSFV. To explore this potential connection, we initially employed mitochondrial probes to label mitochondria in PK-15 and 3D4/2 cells after PKM2 overexpression and observed changes in mitochondrial morphology and number. Laser confocal microscopy images indicated a decrease in the number of mitochondria, shorter length, and increased division in cells overexpressing PKM2 (Fig. 8A). Furthermore, we observed the co-localization of PKM2 with TOM20, a mitochondrial marker protein, confirming PKM2’s localization within the mitochondria (Fig. 8B). Mitophagy is usually associated with decreased mitochondrial quality, and western blot analysis of mitochondrial protein expression is commonly used to assess mitochondrial quality. By detecting autophagy marker proteins LC3, ATG5, and SQSTM1/p62, we found that PKM2 overexpression promoted autophagy. Furthermore, the detection of mitochondrial outer membrane proteins TOM20, COXIV, VDAC1, and lysosomal marker protein LAMP1 revealed a significant increase in mitophagy in PKM2-overexpressed cells compared to control cells (Fig. 8C). Conversely, inhibiting PKM2 yielded the opposite results (Fig. 8D). Transmission electron microscopy (TEM) stands as the gold standard for observing mitophagy. We employed TEM to scrutinize the ultrastructure of mitochondria following the overexpression of PKM2. TEM images revealed that mitochondria in PK-15 and 3D4/2 cells transfected with empty vector p3×Flag-CMV showed typical long tubular structures and had clear mitochondrial cristae. Conversely, the mitochondria in PK-15 and 3D4/2 cells transfected with the Flag-PKM2 plasmid exhibited a departure from the standard mitochondrial cristae structure, and some were wrapped by double or single-layered membrane-like structures, indicative of mitophagy-like structures (Fig. 9A and B; Fig. S1). Quantitative analysis revealed a significant increase in autophagosome-like structures in PKM2-overexpressing cells (Fig. 9C).

The final step of mitophagy involves the binding of phagosomes to lysosomes, facilitating the transport, degradation, and recirculation of mitochondrial substances (45). To discern whether PKM2 induces complete or incomplete autophagy, we transfected a tandem-labeled mRFP-EGFP plasmid encoding a mitochondrial targeting signal sequence into PK-15 and 3D4/2 cells and treated the cells with mitophagy agonist CCCP and mitophagy inhibitor Mdivi-1, respectively. Laser confocal microscopy shows that mitochondria of normal cells appear yellow because they carry both RFP and GFP fluorescence. In contrast, mitochondria presented to lysosomes appear only red due to the burst of GFP fluorescence (46). As demonstrated in Fig. 9D and E, PK-15 and 3D4/2 cells overexpressing PKM2 displayed more red fluorescence compared to the transfected null cells showing yellow fluorescence. This finding was consistent with the CCCP treatment group phenomenon, where Mdivi-1 treatment reversed PKM2-induced mitophagy. These results demonstrated overexpression of PKM2 induced mitochondrial fission and mitophagy.

### Silencing PKM2 inhibited mitophagy induced by CSFV

**Fig 10:**
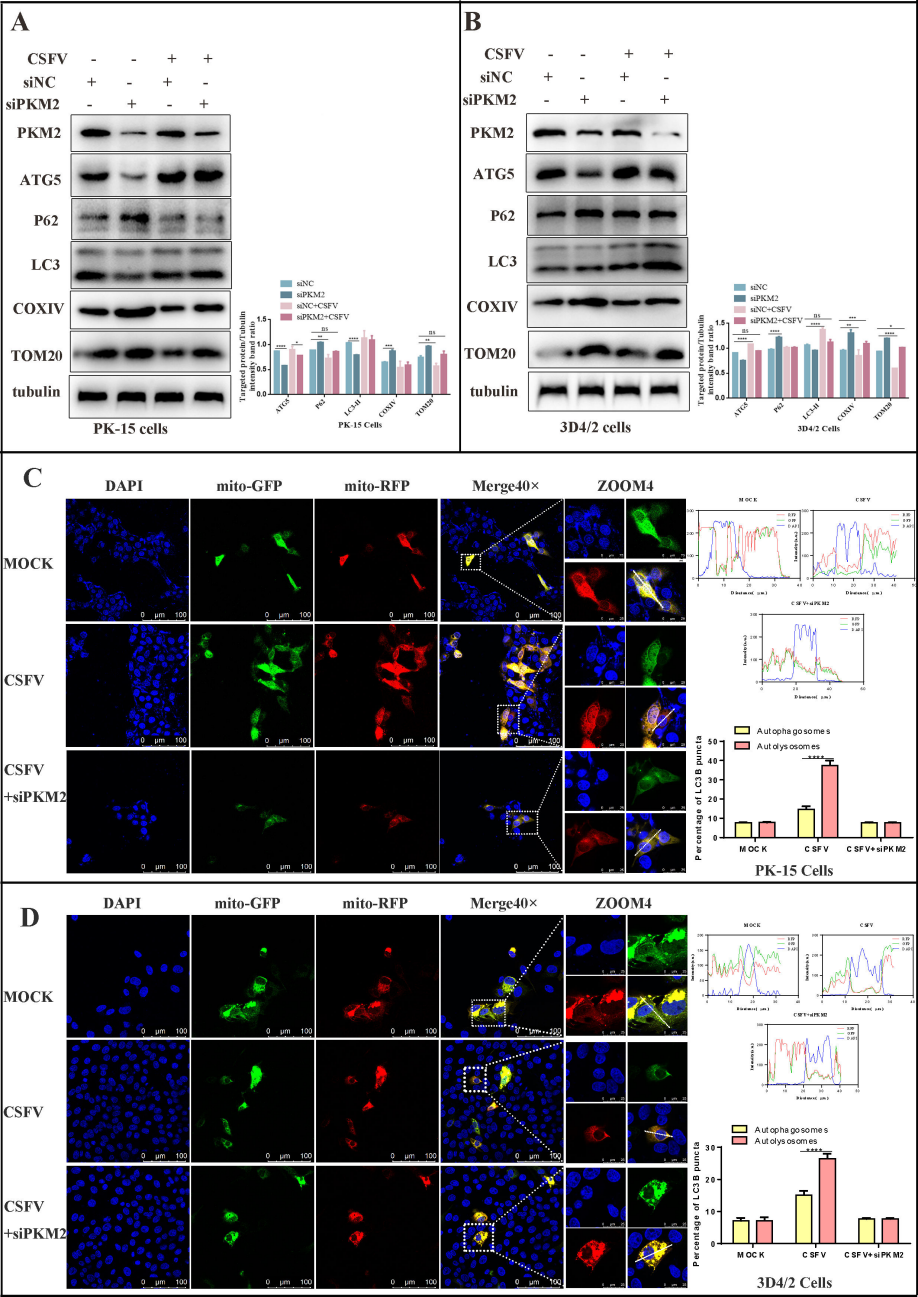
Silencing PKM2 inhibited mitophagy induced by CSFV. (**A and B**) PK-15 (**A**) and 3D4/2 cells (**B**) were transfected with the siRNA of siPKM2 or siNC for 24 h, then mock infected or infected with CSFV (MOI = 1.0). Western blot was used to analyze the relative expression of proteins ATG5, P62, LC3, COXIV, TOM20, and tubulin (loading control). The level of protein was quantified using Image-Pro Plus 6.0 software. Error bars indicate the mean (±SD) of three independent experiments. *P < 0.05; **P < 0.01, and ***P < 0.001 (one-way ANOVA). (**C and D**) PK-15 and 3D4/2 cells transiently expressing Mito-mRFP-EGFP were transfected with siPKM2 or infected with CSFV for 24 h. In the zoomed images, fluorescence signals indicated the expression of mRFP and GFP proteins targeting mitochondria: yellow color, no mitophagy; red color, mitophagy. Image-Pro Plus 6.0 software was used to measure the fluorescence intensity quantitatively. Bar graphs represent the mean number of autophagosomes (puncta with both red and green colors, i.e., puncta with yellow color in merged images) and autolysosomes (puncta with only red but not green color, i.e., puncta with red color in merged images) per cell. Error bars indicate the mean (±SD) of three independent experiments. ns, *P* > 0.05 and ****P < 0.0001 (two-way ANOVA).

Our previous studies have demonstrated that CSFV induces mitochondrial division and mitophagy, which promotes persistent infection. In light of the correlation between PKM2 and mitophagy, we endeavored to unravel the connection between PKM2- and CSFV-induced mitophagy. To accomplish this, we inhibited PKM2 and analyzed the proteins associated with autophagy and mitochondria. We observed that ATG5 and LC3II/LC3I were reduced in PKM2 inhibited cells compared to control cells. Interestingly, CSFV infection reversed the inhibition of mitophagy caused by PKM2 interference (Fig. 10A and B). To further investigate this relationship, we transfected PK-15 and 3D4/2 cells with an mRFP-EGFP plasmid encoding a mitochondrial targeting signal sequence. As depicted in Fig. 10C and D, CSFV-infected PK-15 and 3D4/2 cells displayed an augmented red fluorescence relative to control cells, while CSFV-infected cells with PKM2 inhibition exhibited yellow fluorescence. These results suggest that the inhibition of PKM2 impeded CSFV-induced mitophagy.

### The AMPK-mTOR pathway was upregulated in PKM2-inhibited cells upon CSFV infection

**Fig 11:**
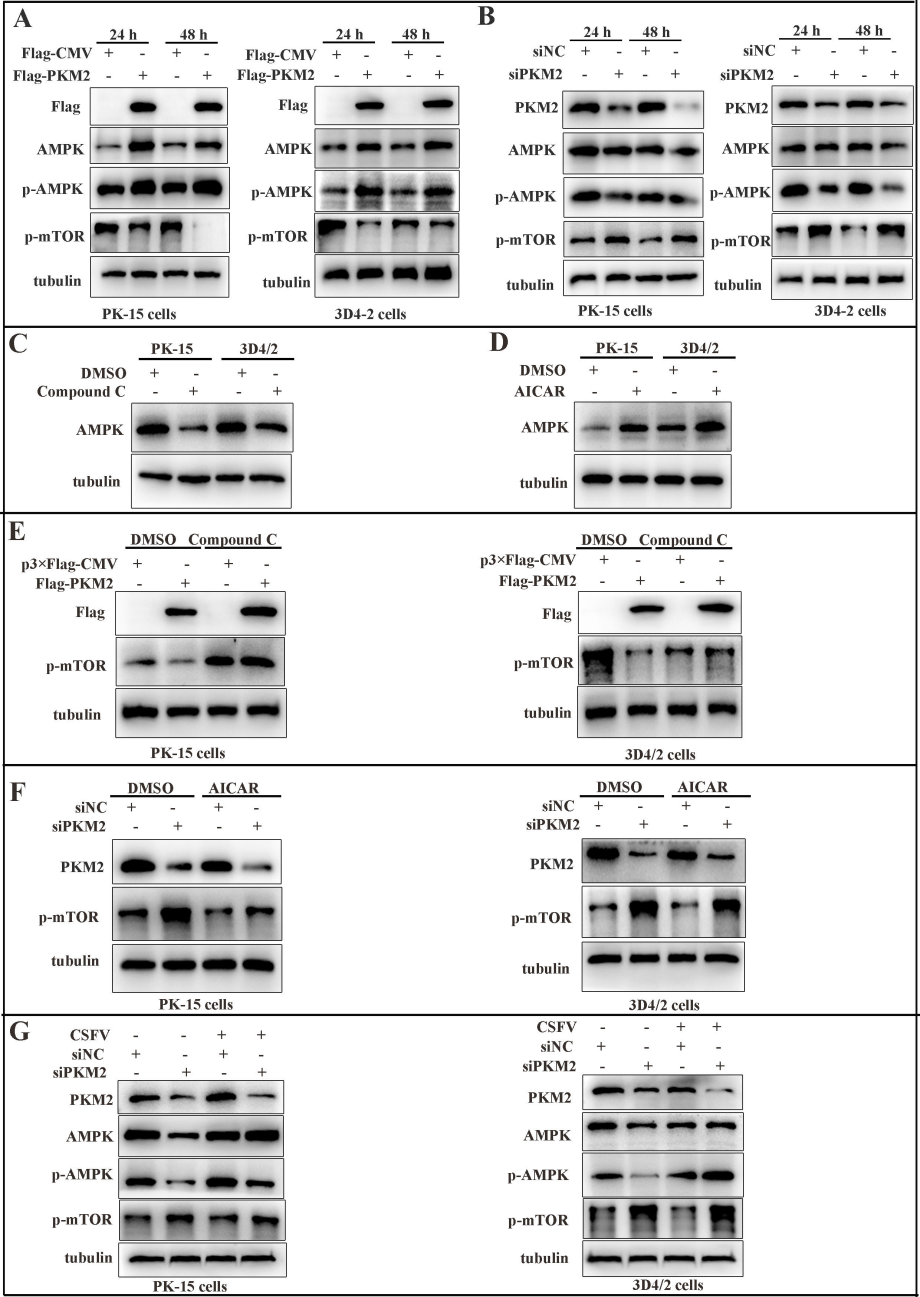
The AMPK-mTOR pathway was upregulated in PKM2-inhibited cells with CSFV infection. (**A and B**) PK-15 and 3D4/2 cells were transfected with p3×Flag-CMV or Flag-PKM2 and (**A**) siPKM2 or siNC (**B**). Cell samples were analyzed at 24 and 48 h by immunoblotting with antibodies against AMPK, p-AMPK, p-mTOR, Flag, PKM2, and tubulin (loading control). (**C and D**) PK-15 and 3D4/2 cells were treated with 10 µM Compound C (**C**) or 10 µM AICAR (**D**). DMSO treatment was used as a control. Cell samples were analyzed at 24 h by immunoblotting with antibodies against AMPK and tubulin (loading control). Bar graphs represent the mean number of autophagosomes (puncta with both red and green colors, i.e., puncta with yellow color in merged images) and autolysosomes (puncta with only red but not green color, i.e., puncta with red color in merged images) per cell. Error bars indicate the mean (±SD) of three independent experiments. ns, *P* > 0.05; ***P < 0.001, and ****P < 0.0001 (two-way ANOVA). (**E**) PK-15 and 3D4/2 cells were treated with DMSO or Compound C and then transfected with p3×Flag-CMV or Flag-PKM2. Cell samples were analyzed at 24 h by immunoblotting with antibodies against Flag, p-mTOR, and tubulin (loading control). (**F**) PK-15 and 3D4/2 cells were treated with 10 µM DMSO or 10 µM AICAR and then transfected with siPKM2 or siNC. Cell samples were analyzed at 24 h by immunoblotting with antibodies against PKM2, p-mTOR, and tubulin (loading control). (**G**) PK-15 and 3D4/2 cells were transfected with the siPKM2 or siNC for 24 h and then mock infected or infected with CSFV (MOI = 1.0). Western blot was used to analyze the relative expression of proteins AMPK, p-AMPK, p-mTOR, and TUBA (loading control).

As a guardian of mitochondrial homeostasis, AMPK plays a pivotal role in mediating the mitochondrial autophagic process through the regulation of downstream signaling pathways. There is growing evidence suggesting that the AMPK-mTOR signaling pathway serves as an essential regulator in triggering autophagic cell death (47). Research has shown that CSFV-mediated autophagy is associated with the inhibition of mTOR phosphorylation (48). To uncover the mechanism underlying PKM2-induced mitophagy, we examined the impact of PKM2 on the AMPK-mTOR signaling pathway. Western blot analysis showed that the overexpression of PKM2 resulted in elevated AMPK and p-AMPK protein expression and decreased p-mTOR expression in PK-15 and 3D4/2 cells compared with control (Fig. 11A). Conversely, the silencing of PKM2 resulted in the opposite (Fig. 11B). To further validate AMPK as the upstream switch of PKM2 regulating the mTOR signaling pathway, cells were treated with the AMPK inhibitor Compound C and the agonist 5-aminoimidazole-4-carboxamide ribonucleoside (AICAR), respectively, with DMSO-treated cells serving as controls. It was found that Compound C significantly suppressed AMPK expression (Fig. 11C), whereas AICAR significantly increased AMPK expression (Fig. 11D). Subsequently, cells were pretreated with DMSO or Compound C for 2 h and then transfected with PKM2. Results showed that Compound C reversed the inhibitory effect of PKM2 on p-mTOR (Fig. 11E). Following this, cells were pretreated with DMSO or AICAR for 2 h and then transfected with PKM2 siRNA. It was observed that AICAR significantly attenuated the promoting effect of siPKM2 on p-mTOR (Fig. 11F). These findings suggested that PKM2 influences the mTOR pathway through AMPK. However, the inhibition of AMPK-mTOR signaling by siPKM2 disappeared when we infected the cells with CSFV at an MOI of 1.0 (Fig. 11G). This demonstrated that CSFV infection reversed the inhibitory effect of siPKM2 on the AMPK-mTOR signaling pathway, and whether CSFV infection activated the AMPK-mTOR signaling pathway through PKM2 remains to be further verified.

### Overexpression of PKM2 promoted mitochondrial enrichment of AMPK

**Fig 12:**
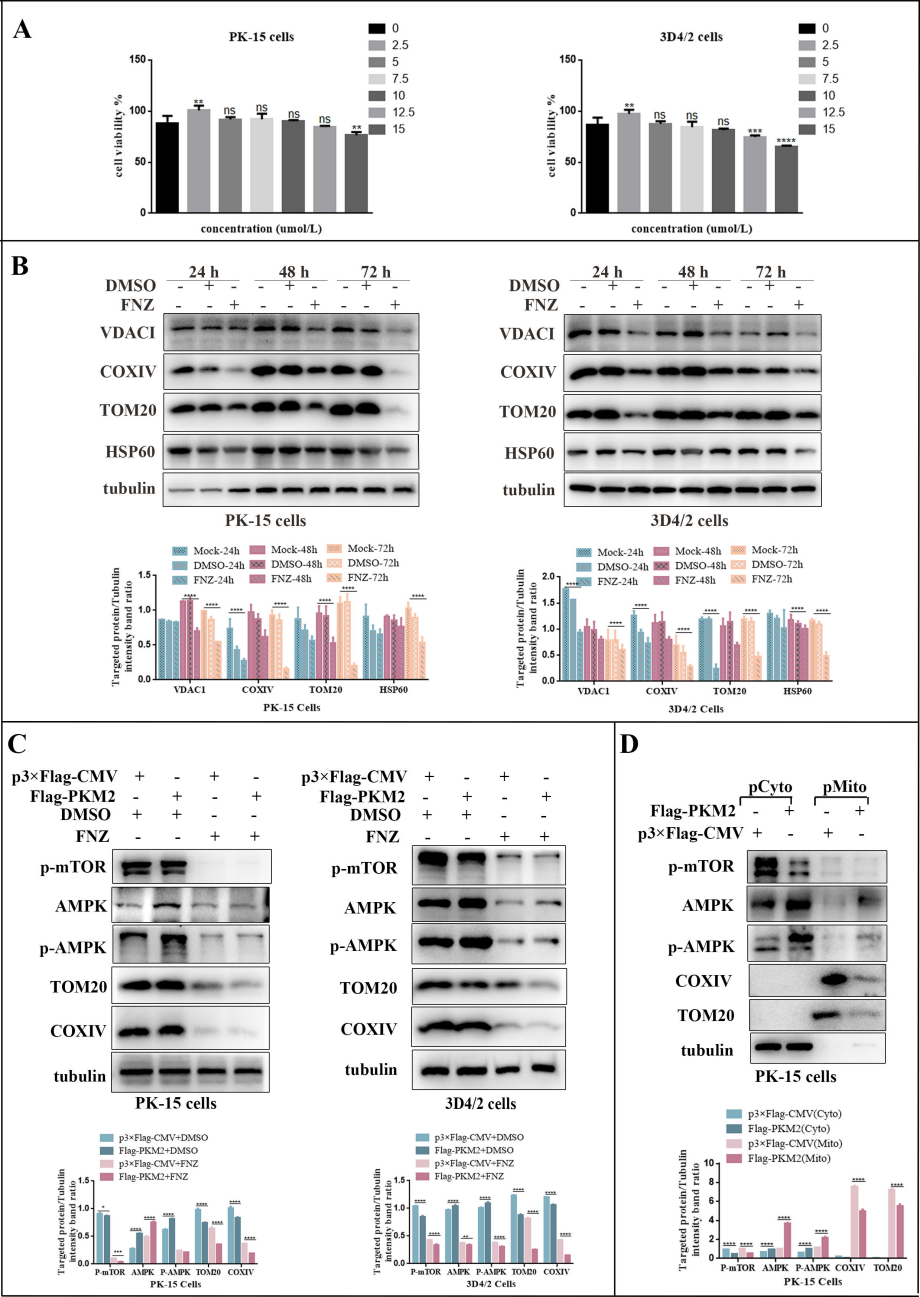
PKM2 activated AMPK and enriched it in mitochondria. (**A**) The effect of FNZ (10 µM) on cell viability of PK-15 and 3D4/2 cells. Cells were treated with different concentrations of FNZ and evaluated by the CCK-8 assay. Error bars represent the mean ± SD; *n* = 3; **P < 0.01; ***P < 0.001; ****P < 0.0001; and ns, *P* > 0.05. (**B**) PK-15 and 3D4/2 cells were treated with FNZ at different times. Western blot was used to analyze the relative expression of VDACI, COXIV, TOM20, HSP60, and tubulin (loading control). (**C**) PK-15 and 3D4/2 cells were transfected with p3×Flag-CMV or Flag-PKM2 and then treated with 10 µM FNZ, the same volume of DMSO. Western blot was used to analyze the relative expression of proteins AMPK, p-AMPK, p-mTOR, TOM20, COXIV, and tubulin (loading control). (**D**) Effect of PKM2 on AMPK mitochondrial translocation. PK-15 cells were transfected with P3×Flag-CMV or Flag-PKM2, followed by mitochondrial isolation using the Mitochondrial Isolation Kit. Western blot was used to analyze the relative expression of proteins AMPK, p-AMPK, p-mTOR, TOM20, COXIV, and tubulin (loading control). The level of protein was quantified using Image-Pro Plus 6.0 software. Error bars indicate the mean (±SD) of three independent experiments. *P < 0.05; **P < 0.01; ***P < 0.001; and ****P < 0.0001 (one-way ANOVA).

To investigate the precise localization of AMPK activation by PKM2, we employed flunarizine (FNZ) to eliminate mitochondria, as it has been reported to induce mitochondrial dysfunction and reduce mitochondrial mass (49). To ensure that FNZ did not affect host cell viability or alter mitochondrial mass, we initially assessed the effect of various concentrations of FNZ on the viability of PK-15 and 3D4/2 cells using the CCK-8 assay. At a final concentration of 10 µmol/L, FNZ was found to be non-cytotoxic (Fig. 12A). Subsequently, we monitored the mitochondrial mass in PK-15 and 3D4/2 cells using 10 µM of FNZ. Treatment with FNZ for 72 h resulted in a significant time-dependent reduction of TOM20, VDAC, COXIV, and matrix proteins HSP60 (Fig. 12B). Therefore, we selected this concentration for further investigation. Our results demonstrated that overexpression of PKM2 had little effect on AMPK and p-mTOR expression in mitochondria-removed cells after mitochondria elimination using FNZ, while promoting the protein expression of AMPK and p-mTOR in normal cells (Fig. 12C). We speculated that PKM2 may be responsible for the mitochondrial enrichment of AMPK. To investigate this possibility, we isolated and purified mitochondria from PK-15 cells that overexpressed PKM2. We then examined the extent of enrichment of AMPK, p-AMPK, and p-mTOR protein molecules in the purified mitochondria using western blot. The results indicated that PKM2 promoted the expression of AMPK and p-AMPK in mitochondria (Fig. 12D). These results suggested that overexpression of PKM2 caused enrichment of AMPK in mitochondria. However, whether this is related to PKM2-induced mitophagy needs to be further explored.

### PKM2 mediated mitophagy by activating the AMPK-mTOR pathway

**Fig 13:**
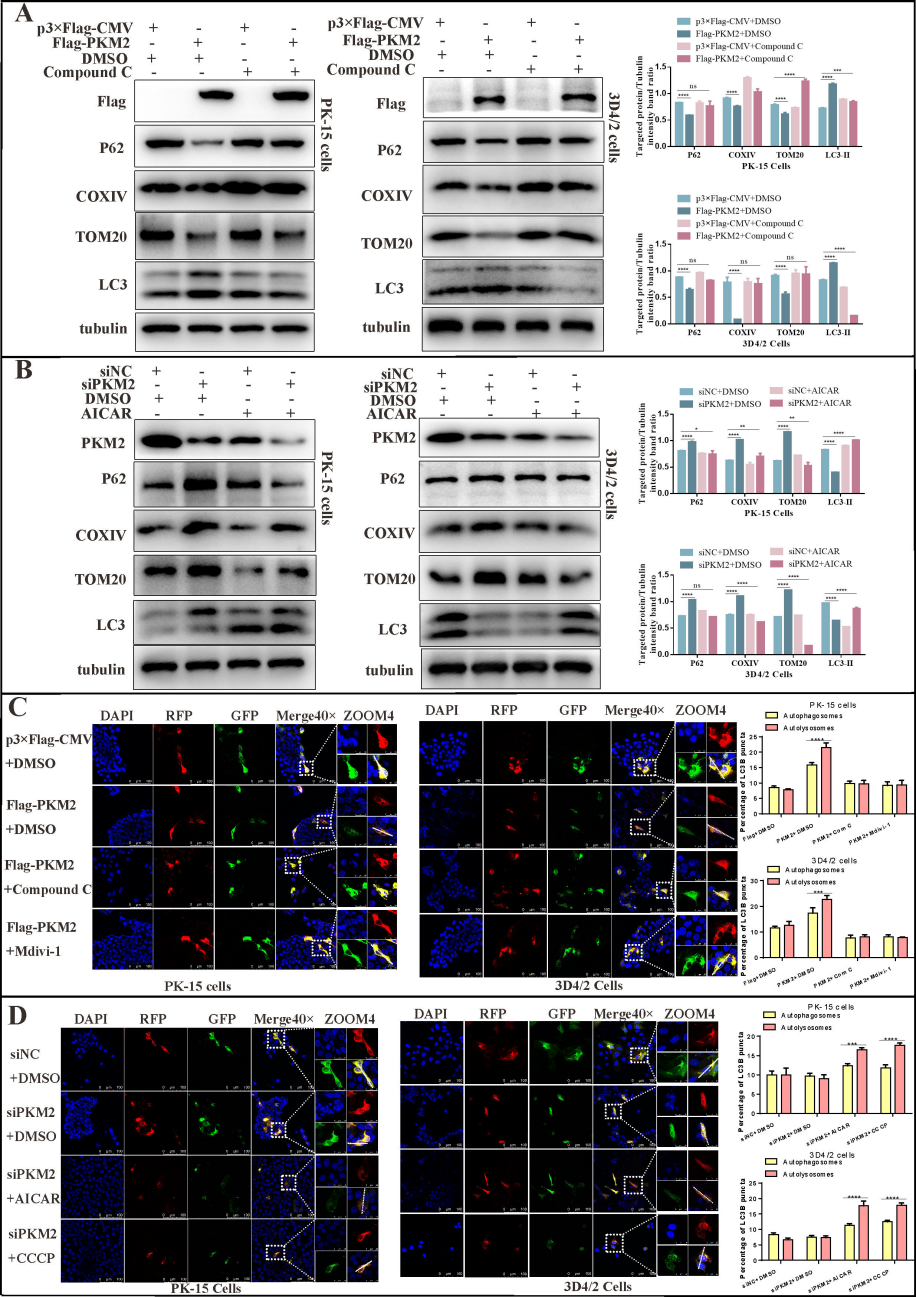
PKM2 regulated mitophagy through the AMPK-mTOR pathway. (**A**) PK-15 or 3D4/2 cells were pretreated with DMSO or Compound C (10 µM) for 2 h and then transfected with p3×Flag-CMV or Flag-PKM2. Cell samples were analyzed at 24 h by immunoblotting with antibodies against Flag, P62, COXIV, TOM20, LC3, and tubulin (loading control). (**B**) PK-15 or 3D4/2 cells were pretreated with DMSO or AICAR for 2 h and then transfected with siNC or siPKM2. Cell samples were analyzed at 24 h by immunoblotting with antibodies against PKM2, P62, COXIV, TOM20, LC3, and tubulin (loading control). The level of protein was quantified using Image-Pro Plus 6.0 software. Error bars indicate the mean (±SD) of three independent experiments. *P < 0.05; **P < 0.01; ***P < 0.001; and ****P < 0.0001 (one-way ANOVA). (**C**) PK-15 and 3D4/2 cells transiently expressing Mito-mRFP-EGFP were pretreated with DMSO/Compound C/Mdivi-1 (10 µM) for 2 h and then transfected with p3×Flag-CMV or Flag-PKM2. In the zoomed images, fluorescence signals indicated the expression of mRFP and GFP protein targeting mitochondria: yellow color, no mitophagy; red color, mitophagy. (**D**) PK-15 and 3D4/2 cells transiently expressing Mito-mRFP-EGFP were pretreated with DMSO/AICAR/CCCP (10 µM) for 2 h and then transfected with siNC or siPKM2. In the zoomed images, fluorescence signals indicated the expression of mRFP and GFP proteins targeting mitochondria: yellow color, no mitophagy; red color, mitophagy. Image-Pro Plus 6.0 software was used to measure the fluorescence intensity quantitatively. ns, *P* > 0.05; ***P < 0.001; and ****P < 0.0001 (two-way ANOVA).

Given the critical role of AMPK activation in mitophagy and our previous finding that PKM2 overexpression induced mitophagy (Fig. 9), we investigated whether PKM2 regulates mitophagy via the AMPK-mTOR axis. To this end, we pretreated cells with either DMSO or Compound C for 2 h and then transfected PKM2 plasmids. Western blot revealed that Compound C elevated the levels of TOM20, COXIV, and P62 proteins and significantly attenuated the activation of mitophagy by PKM2 overexpression compared to the control (Fig. 13A). Additionally, cells were pretreated with DMSO or AICAR for 2 h and then transfected with PKM2 siRNA. The results indicated that AICAR decreased the protein levels of TOM20, COXIV, and p62 while reversing the inhibitory effect of siPKM2 on mitophagy (Fig. 13B). Furthermore, the Mito-GFP-RFP dual fluorescent reporter system was analyzed using laser confocal microscopy. The images showed a partial burst of GFP fluorescence with a high percentage of red fluorescence in cells overexpressing PKM2. In cells treated with Compound C, GFP and RFP fused into a typical yellow color, consistent with the results of the Mdivi-1 treatment group (Fig. 13C). The AICAR and CCCP treatment groups reversed the inhibition of mitophagy due to the silencing of PKM2 (Fig. 13D) (Quantitative analysis of the fluorescence intensity of zoom4 images is shown in Fig. S2.). These results suggested that PKM2-mediated mitophagy occurs through the AMPK-mTOR signaling pathway.

### PKM2 promoted CSFV proliferation via AMPK

**Fig 14:**
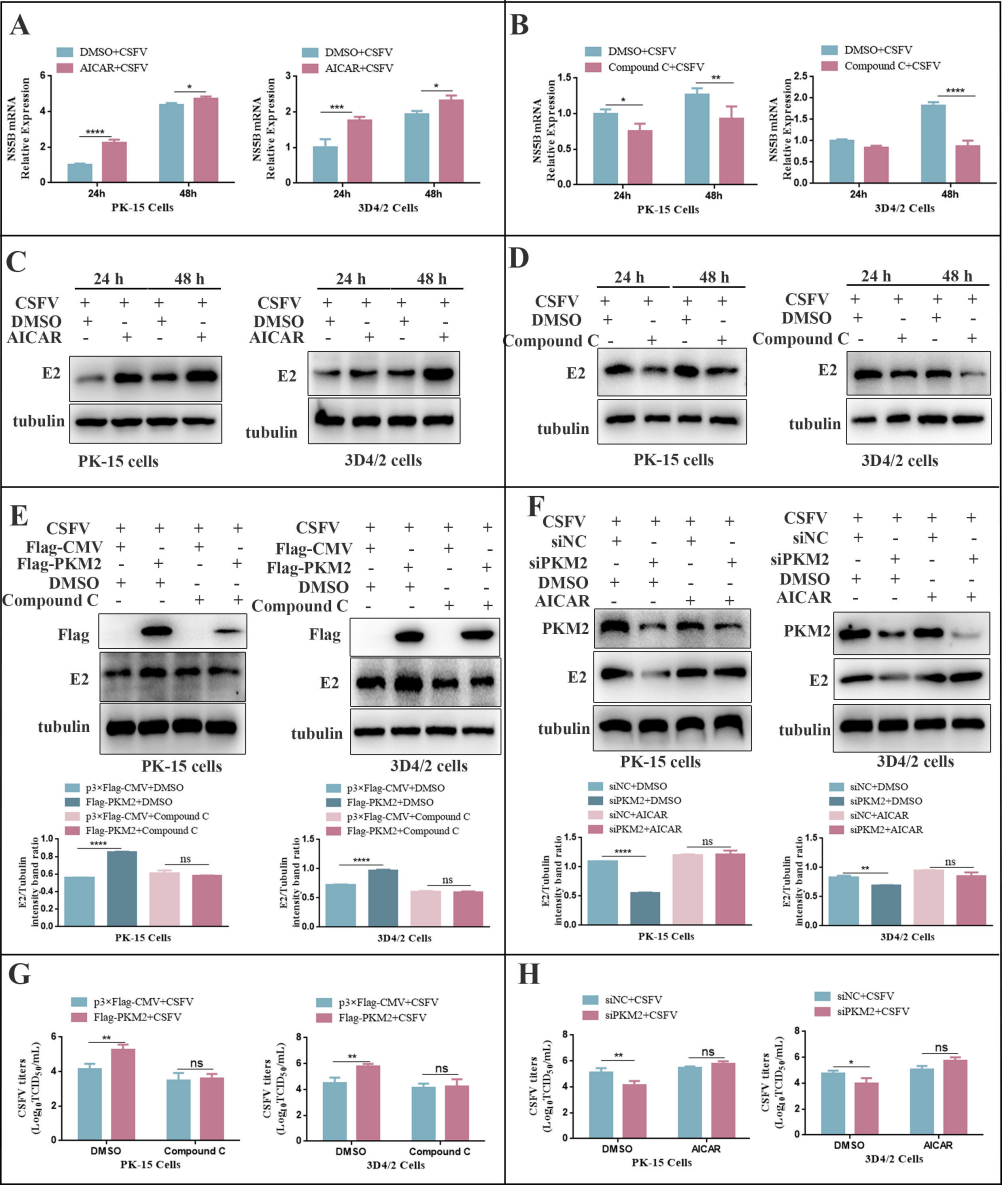
PKM2 promoted CSFV proliferation via AMPK. (A and B) PK-15 or 3D4/2 cells were infected with CSFV (MOI = 1.0) for 2 h and treated with 10 µM AICAR (**A**) or Compound C (**B**), the same volume of DMSO. The relative expression of NS5B mRNA was detected by qRT-PCR at 24 h. (**C and D**) Cell treatment is the same as panel A. Cell samples were analyzed at 24 h by immunoblotting with antibodies against E2 and tubulin (loading control). (**E**) PK-15 or 3D4/2 cells were pretreated with DMSO or Compound C (10 µM) for 2 h and then transfected with p3×Flag-CMV or Flag-PKM2. Cell samples were analyzed at 24 h by immunoblotting with antibodies against Flag, E2, and tubulin (loading control). (**F**) PK-15 or 3D4/2 cells were pretreated with DMSO or AICAR (10 µM) for 2 h and then transfected with siNC or siPKM2. Cell samples were analyzed at 24 h by immunoblotting with antibodies against PKM2, E2, and tubulin (loading control). The level of protein was quantified using Image-Pro Plus 6.0 software. Error bars indicate the mean (±SD) of three independent experiments. ns, *P* > 0.05; *P < 0.05; **P < 0.01; ***P < 0.001; and ****P < 0.0001 (one-way ANOVA). (**G**) Cell treatment is the same as panel **E**. CSFV virus titers in the supernatant were determined as 50% tissue culture infective doses (TCID50)/mL as described in Materials and Methods. (**H**) Cell treatment is the same as panel **F**. CSFV virus titers in the supernatant were determined as TCID50/mL as described in Materials and Methods. Error bars indicate the mean (±SD) of three independent experiments. ns, *P* > 0.05; *P < 0.05; **P < 0.01; and ***P < 0.001 (one-way ANOVA).

To investigate the effect of AMPK on CSFV replication, we infected PK-15 and 3D4/2 cells with AICAR or Compound C for 24 and 48 h, followed by the analysis of NS5B gene and E2 protein expression using RT-qPCR and western blot. Our findings revealed that AICAR significantly increased the relative expression of the NS5B gene and E2 protein compared to the control group (Fig. 14A and C), whereas Compound C significantly decreased their expression (Fig. 14B and D). To further determine whether the promoting effect of PKM2 on CSFV proliferation is associated with AMPK activation, we treated PK-15 and 3D4/2 cells with Compound C or AICAR before transfecting PKM2 or siPKM2. After 24 h of CSFV infection, we detected E2 protein expression and virus titer. Our results indicated that Compound C treatment weakened the promoting effect of overexpressed PKM2 on CSFV proliferation (Fig. 14E and G), while AICAR reversed the inhibitory effect of siPKM2 on CSFV proliferation (Fig. 14F and H), implying that PKM2 promoted CSFV proliferation through AMPK activation.

### PKM2 promoted CSFV proliferation through mitophagy

**Fig 15:**
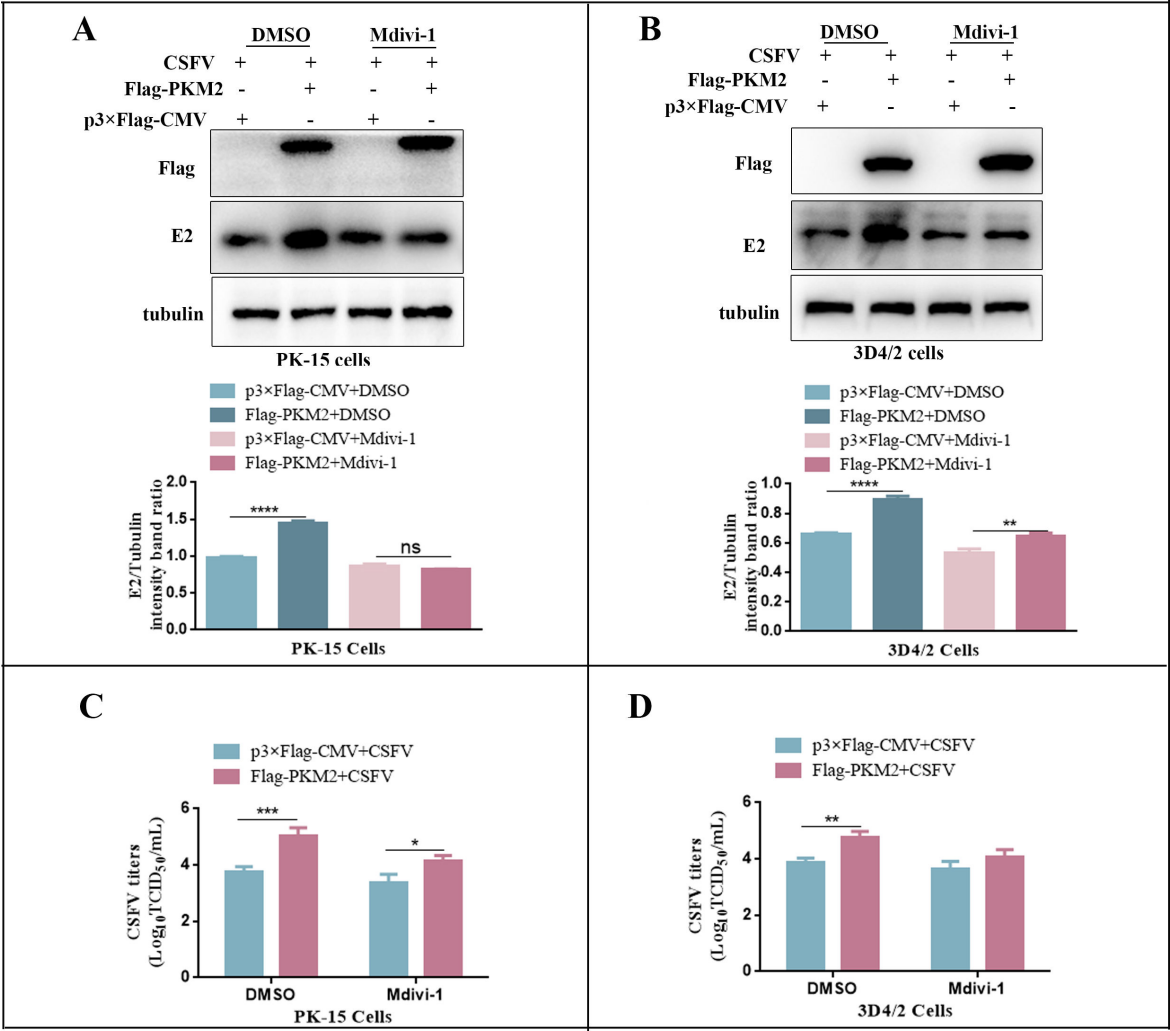
Overexpression of PKM2 promoted CSFV proliferation via mitophagy. (**A and B**) PK-15 or 3D4/2 cells were infected with CSFV (MOI = 1.0) for 2 h and treated with 10 µM Mdivi-1, the same volume of DMSO. Cell samples were analyzed at 24 h by immunoblotting with antibodies against E2 and tubulin (loading control). The level of protein was quantified using Image-Pro Plus 6.0 software. Error bars indicate the mean (±SD) of three independent experiments. *P < 0.05; **P < 0.01; ***P < 0.001; and ****P < 0.0001 (one-way ANOVA). (**C and D**) Cell treatment is the same as panel A. CSFV virus titers in the supernatant were determined as 50% tissue culture infective doses (TCID50)/mL as described in Materials and Methods. Error bars represent the mean ± SD; *n* = 3; *P < 0.05; **P < 0.01; ***P < 0.001; and ns, *P* > 0.05 (one-way ANOVA).

To further explore the relationship between PKM2-induced mitophagy and CSFV proliferation, we treated PK-15 and 3D4/2 cells with the mitophagy inhibitor Mdivi-1 prior to transfection with PKM2. After 24 h of CSFV infection, we analyzed the expression of the E2 protein and virus titer. It was found that overexpression of PKM2 increased the expression of the E2 protein. However, the promotion effect of PKM2 on E2 protein expression was abolished after treatment with Mdivi-1 (Fig. 15A and B). Similarly, Mdivi-1 inhibited the promotion effect of PKM2 overexpression on virus titer (Fig. 15C and D), indicating that PKM2 promoted CSFV proliferation through the mitophagy pathway.

### Cell viability was not affected by RNA interference

**Fig 16:**
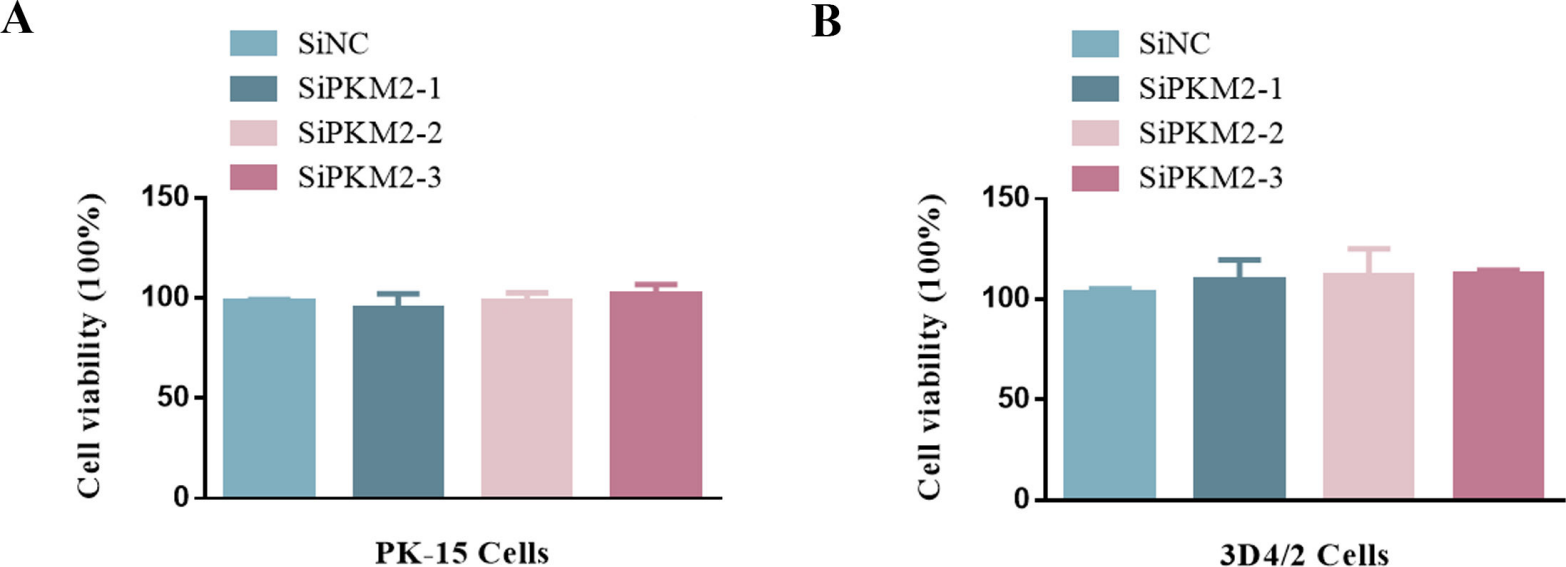
The effect of RNA interference on cell viability. The cell viability of PK-15 (A) cells and 3D4/2 (B) cells transfected with siNC or PKM2 siRNA-1/-2/-3 was analyzed using the CCK8 assay as described in Materials and Methods (mean ± SD; *n* = 3; ns, *P* > 0.05).

To exclude the possibility that PKM2 siRNA affected CSFV replication, we determined the effects of RNA interference on the viability of PK-15 and 3D4/2 cells. No significant change in cell viability was observed following the knockdown of the PKM2 (Fig. 16).

## DISCUSSION

Viruses rely on the host cell’s material-energy metabolic system for replication (50), leading to host metabolic disorders and subsequent immunosuppression (8), which is considered a major cause of persistent viral infections. However, many viruses have developed replication strategies to adapt to unfavorable host environments. Metabolomic studies have revealed that viral infections can reshape host glycolytic metabolic pathways and affect metabolite levels. For instance, NDV infection reprograms cellular metabolism by increasing glucose utilization in the glycolytic pathway, and SARS-CoV-2 infection upregulates the expression of multiple metabolites, including glucose transporter protein 1, lactate dehydrogenase (LDH), and pyruvate dehydrogenase kinase 1 (51, 52). Our previous studies have demonstrated that CSFV infection remodels host cell glycolytic metabolism, facilitating viral replication (53).

The “Warburg effect” is a hallmark of cancer cells, where they produce a large amount of lactic acid and consume more energy under aerobic conditions, and PKM2 plays a crucial role in the last stage of this process to stimulate cancer cell growth. Evidence suggests that many metabolic regulatory enzymes act as signaling molecules in viral infection and immune regulation (54). For example, HIF-1α promotes aerobic glycolysis in endothelial cells infected with Kaposi’s sarcoma herpesvirus through upregulation of PKM2 expression (55). Similarly, African swine fever virus infection inhibits IFN-β expression through LDH-induced elevation of lactate levels, thereby favoring self-replication (56). However, the role of PKM2 in CSFV infections has not been clearly characterized. In this study, we found that CSFV infection promoted PKM2 expression both *in vitro* and *in vivo* (Fig. 1). Pyruvate is the end product of glycolysis and is ultimately translocated to the mitochondria. Our study showed that during CSFV infection, pyruvate levels decline. Simultaneously, CSFV infection prompts an upregulation of PKM2 expression while impairing its capacity to augment pyruvate levels (Fig. 2). This phenomenon likely arises due to the intricate nature of pyruvate metabolism regulation within the organism. Mutations affecting the genes responsible for encoding regulatory enzymes and disruptions at pivotal junctures of pyruvate metabolism may both contribute to the development of disease (57). Furthermore, elevated pyruvate levels may disrupt metabolic pathways like glucose metabolism, fatty acid metabolism, and amino acid metabolism. Consequently, the cell initiates a series of responses to counteract PKM2’s ability to elevate pyruvate levels following CSFV infection, all in an effort to maintain physiological equilibrium. Given that pyruvate is a direct metabolite of pyruvate kinase, we speculate that PKM2 may play a significant role in CSFV infection.

It often evades the immune response during viral infection by interacting with host proteins. For example, pseudorabies virus (PRV) pUL16 assists the nuclear import of VP26 through protein-protein interactions (58). Interactions between different proteins in various virus components provide favorable conditions for viral replication. For instance, the interaction between capsid protein VP5 and periplasmic proteins of PRV (pUL36, pUL37, and pUL36) facilitates the movement of the nucleocapsid to the Golgi for further processing (59). A previous study showed that NS3, NS4A, NS4B, NS5A, and NS5B are required for CSFV replication (60). Interaction with host cell proteins is necessary for the life cycle of CSFV, and cellular beta-actin interacts with the E2 protein to affect the early stages of the CSFV replication cycle (61). Moreover, the interaction of Anx2 and NS5A enhances viral assembly rather than genome replication and viral particle release (62). Heat shock protein 70 (63) and eukaryotic elongation factor 1A (64) have also been shown to interact with NS5A of CSFV to promote viral RNA replication. Here, we found that PKM2 interacted with the CSFV non-structural proteins NS4A and NS5A and promoted their expression (Fig. 3 and 4). We also observed that overexpression of PKM2 promoted CSFV replication, whereas silencing of PKM2 inhibited CSFV replication (Fig. 5). *In vitro*, pyruvate supplementation has been demonstrated to prevent cell death and support fetal development in cases of Zika virus infection (65). Similarly, we found that pyruvate supplementation *in vitro* promoted the expression of CSFV N^pro^ protein and had a rescue effect on the inhibition of CSFV proliferation caused by PKM2 knockdown (Fig. 6).

A growing number of studies have shown that pyruvate metabolism is closely related to mitochondrial biological functions (66, 67). Furthermore, PKM2 interacted with MFN2, a key regulator of mitochondrial fusion, thereby promoting mitochondrial fusion and oxidative phosphorylation and attenuating glycolysis (14). It has also been reported that PKM2 translocated to mitochondria in response to oxidative stress and regulated oxidative stress-induced apoptosis by phosphorylating Bcl2 (21). Furthermore, PKM2 promoted VR-EPC angiogenesis by regulating glycolysis, mitochondrial division, and fusion (68). In this study, we found that PKM2 caused a high production of ROS and a decrease in membrane potential in mitochondria (Fig. 7). Mitochondrial quality control is essential for protecting cells from adverse environmental effects and is mainly regulated by mitophagy (69, 70). Our results showed that PKM2 overexpression was followed by an increase in the mitochondrial division and a decrease in the expression of mitochondrial membrane proteins (Fig. 8). TEM results revealed that the length of mitochondria became shorter and elliptical, and the mitochondrial crest disappeared obviously after PKM2 overexpression (Fig. 9A and B). Additionally, we utilized the dual fluorescence reporter (mito-mRFP-EGFP) to analyze mitophagy in PKM2 overexpression cells (Fig. 9D and E). The inhibition of PKM2 impeded mitophagy induced by CSFV infection (Fig. 10). Our findings indicate that CSFV infection in PK-15 cells exhibits greater sensitivity to the reversal of autophagy inhibition induced by PKM2 interference. This heightened sensitivity may be attributed, in part, to the distinct types of PK-15 cells (epithelial cells) and 3D4/2 cells (macrophage), which could result in variations in the expression levels of autophagy-related genes. Notably, some autophagy-related genes may display increased activity in PK-15 cells, thereby promoting autophagy. Furthermore, PK-15 cells might have a proclivity to rely on autophagy to meet their energy requirements under specific conditions.

Autophagy is an evolutionary conserved cellular process serving to degrade cytosolic organelles or foreign material to maintain cellular homeostasis. It has been shown that some members of the *Flaviviridae* family can utilize autophagy mechanisms to promote replication, including HCV and varicella zoster virus (71, 72). mTORC1 is the primary regulatory complex of autophagy and negatively regulates the process (73, 74). Previous studies have shown that the AKT-mTOR, AMPK-mTOR, MAPK1/3-mTOR, and CAMKK2-PRKAA-mTOR pathways are activated after CSFV infection. It was demonstrated that PKM2 typically forms a complex with AMPK to function in cancer cells. For example, nuclear AMPK promotes the transcription of genes associated with cell migration by recruiting PKM2. Nuclear AMPK promotes transcriptional activation of genes involved in cell migration by recruiting PKM2. Nuclear translocation of the PKM2/AMPK complex sustains cancer stem cell populations under glucose-deprived conditions (75). To clarify the potential mechanism of PKM2-induced mitophagy, we analyzed the effect of PKM2 on the AMPK-mTOR signaling pathway in PK-15 and 3D4/2 cells. Our findings revealed that overexpression of PKM2 activated the AMPK-mTOR signaling pathway, and CSFV infection attenuated the inhibitory effect of siPKM2 on the AMPK-mTOR signaling pathway (Fig. 11). The observed reversal effect exhibited a heightened prominence within PK-15 cells. Previous investigations have illuminated the induction of autophagy in PK-15 cells by porcine circovirus through the mediation of the AMPK/mTOR pathway (76). We hypothesized that the heightened sensitivity in PK-15 cells to the AMPK-MTOR pathway could potentially be attributed to their elevated AMPK activity or diminished mTOR activity.

Considering that AMPK exerts its function through translocation (77), we further examined AMPK expression using the mitochondrial elimination drug FNZ and isolated purified mitochondria. It was found that overexpression of PKM2 promoted enrichment of AMPK in mitochondria (Fig. 12). In order to clarify the role of PKM2-induced mitophagy in CSFV replication and provide a basis for further research on the relationship between mitophagy and immune escape from CSFV, we investigated the effect of AMPK on CSFV replication. By using the AMPK agonist AICAR and AMPK inhibitor Compound C, we found that AMPK activation promoted CSFV replication and that PKM2 promoted mitophagy and CSFV proliferation through the activation of AMPK (Fig. 13 and 14).

Additionally, studies have demonstrated that mitophagy can be regulated by the PINK-Parkin and Drp1 pathways. Hence, in this study, we used CCCP and Mdivi-1 to activate and inhibit mitophagy, respectively, and found that PKM2 affected CSFV proliferation by activating AMPK-induced mitophagy (Fig. 15). Previous studies have shown that CSFV replication is required for the induction of autophagy, and autophagosomes are also involved in viral replication and maturation (33). Similarly, PKM2-induced mitophagosome-like vesicle membranes are also likely to provide replication sites for CSFV. The relationship between PKM2-induced AMPK mitochondrial enrichment and mitophagy warrants further exploration.

**Fig 17:**
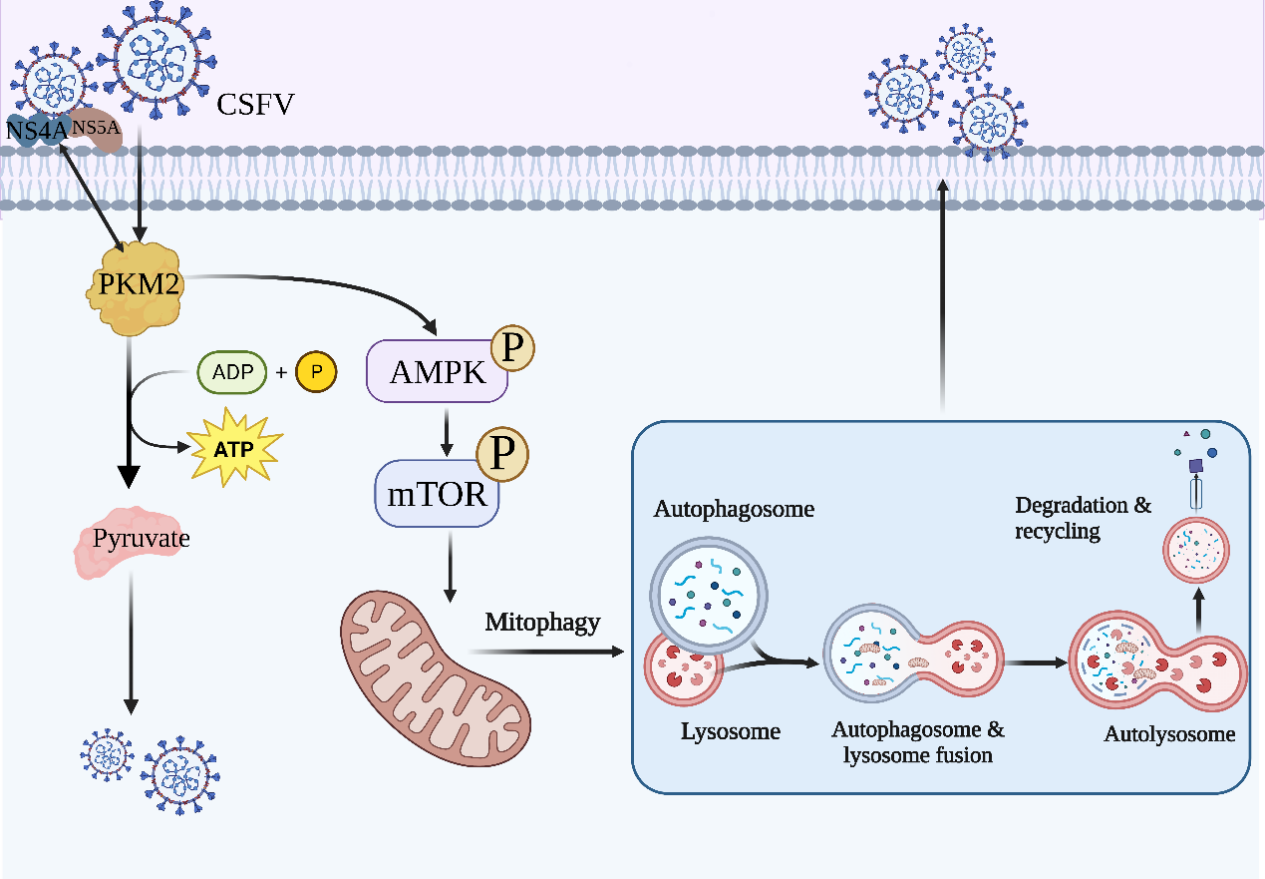
A model for PKM2 promoted CSFV proliferation by inducing mitophagy via the AMPK-mTOR signaling pathway. Image created with biorender.com.

In summary, our study has highlighted the crucial role of the metabolic enzyme PKM2 in host cell mitophagy via the AMPK-mTOR signaling pathway and promoting CSFV infection by inducing mitophagy. Our findings shed light on the mechanisms underlying the regulation of CSFV replication by metabolic pathway enzymes, providing a theoretical basis for the prevention and control of this infection (Fig. 17). These results not only provide a better understanding of the complex interplay between metabolism and CSFV infection but also identify potential therapeutic targets for future research.

## MATERIALS AND METHODS

### Antibodies, plasmids, and chemicals

The primary antibodies used in the study were specific for LC3B (Cell Signaling Technology, 2775), ATG5 (Beyotim, AF2269), BECN1 (Cell Signaling Technology, 3495), LAMP1 (Beyotim, AF7353), VDACI (Beyotim, AF1027), COXIV (Beyotim, AF6549), TOM20 (Beyotim, AF1717), SQSTM1/p62 (Cell Signaling Technology, 23214), HSP60 (Beyotim, AF1771), AMPK (Beyotim, AF1627), p-AMPK (Beyotim, AA393), p-mTOR (Beyotim, AF5869), PKM2 (Santa Cruz Biotechnology, sc-365684), GAPDH (Beyotime, AG019), Tubulin (Beyotime, AT819), mouse monoclonal anti-Flag (Sigma-Aldrich, F7425), and Mouse anti-GFP antibody (AG281). The secondary antibodies used for immunoblotting analysis were HRP-conjugated goat anti-mouse IgG (Bioworld Technology, BS12478), HRP-conjugated goat anti-rabbit IgG (Bioworld Technology, BS13278), and HRP-conjugated rabbit anti-goat IgG (Bioworld Technology, BS30503). The secondary antibodies used for immunofluorescence were Alexa Fluor 350 goat anti-mouse IgG (Beyotime, A0412), Alexa Fluor 488 goat anti-mouse IgG (Beyotime, A0428), and Alexa Fluor 647 goat anti-rabbit IgG (Beyotime, A0468). CSFV N^pro^ and E2 antibodies were prepared in our laboratory. pEGFP-NS4A, pEGFP-NS5A, pEGFP-C1, mito-eGFP-RFP-lc3, and pCMV-3×Flag were prepared in our laboratory. Additionally, three siRNAs targeting different sites within the coding sequences of PKM2, along with the scrambled siRNA, were designed and obtained from Sangon Biotech. Carbonyl cyanide m-chlorobenzyl hydrazone, 3-dihydroxy-2-thio-4 (1H)–quinazolinone (Mdivi-1), and 4′,6-diamidino-2-phenylindole (C1005) were purchased from Beyotim. 5-Aminoimidazole-4-carboxamide ribonucleoside, Compound C, and pyruvic acid (PA) were purchased from Med Chem Express. Viral RNA was extracted using the TaKaRa MiniBEST Viral RNA/DNA Extraction Kit version 5.0 (TaKaRa, 9766), and cDNA synthesis was performed using the PrimeScriptTM II First-Strand cDNA Synthesis Kit (TaKaRa, 6210A) according to the manufacturer’s protocol.

### Cells and virus

The swine kidney cell line PK-15 (ATCC, CCL-33) was cultured in complete DMEM (Gibco, C11995500BT) supplemented with 10% fetal bovine serum (FBS) (Gibco, 10099), and 1% penicillin/streptomycin (Gibco, 15140-122). The porcine macrophage cell line 3D4/2 (ATCC, CRL-2845) was maintained in RPMI 1640 medium (Gibco, C11875500BT) containing 10% FBS and 1% antibiotics. The cells were incubated at 37°C with 5% CO_2_. The CSFV strain (Shimen) used in this study was prepared in our laboratory. To determine the virus titer, we cultured cells in 96-well plates inoculated with 10-fold series diluted virus and incubated at 37°C for 2 days. Cells were immobilized at 37°C with 80% acetone for 30 min at −20°C, and the virus was detected by immunofluorescence using mouse anti-CSFV E2 antibody and goat anti-mouse secondary antibody bound to FITC. The virus titer was calculated according to Karber method and expressed as 50% tissue culture infection dose per mL (TCID50). The multiplicity of infection was determined from the viral titers of each cell line.

### Viral infection

PK-15 and 3D4/2 cells were grown to approximately 80% confluence in cell culture plates and were infected with CSFV at an MOI of . After 2 h, the inoculum was removed by aspiration. The cells were then washed with sterile phosphate-buffered saline (PBS, Thermo Fisher Scientific, 10010023) and incubated in fresh medium at 37°C for the indicated times until harvesting. The mock was infected with phosphate-buffered saline.

### DNA constructs and RNA interference

The full-length swine PKM2 gene (GenBank, XP 001929104) was amplified by PCR and cloned into the p3×Flag-CMV vector using EcoRI (TaKaRa, 1611) and Bgl II (TaKaRa, 1606) to generate p3×Flag-PKM2. The primers used for PKM2 genes are listed in Table 1. Sangon Biotech synthesized siRNAs against PKM2 and AMPK. siRNA sequences are listed in Table 2. PK-15 or 3D4/2 cells were grown to 60%–70% confluence in 12-well cell culture plates and were transfected with siRNAs and plasmid using Lipofectamine 3000 reagent (ThermoFisher, L3000015) according to the manufacturer’s instructions. Briefly, 2 µg of plasmid or 50 nM siRNA and 3 µL P3000 were diluted in 50 µL of serum-free OptiMEM (ThermoFisher Scientific, 22600050), and 3 µL Lipofectamine 3000 was also diluted in 50 µL of serum-free OptiMEM. The dilutions were mixed thoroughly and incubated at 25°C for 10–15 min. The mixture was pipetted into the OptiMEM and cultured at 37°C for 24 h. Following CSFV infection, the cells were incubated in a fresh medium at 37°C for 48 h. The protein targeted for overexpression or knockdown was evaluated by western blot.

**TABLE 1:** Primers used in this study

Gene	Sequence (5′–3′)	GenBank accession no.
PKM2	F: CGGAATTCATGCCGAAGCCCCACAGTGA R: GAAGATCTTCACGGCACAGGCACTACGC	XP 001929104
Q-PKM2	F: GGCTCGTGGTGATCTAGGCATTG R: GTGGCACAGATGACAGGCTTCC	XP 001929104
Q-CSFV-NS5B	F: CCTGAGGACCAAACACATGTTG R: TGGTGGAAGTTGGTTGTGTCTG	MW822569.1
Q-IFNα	F:CTCAGCCAGGACAGAAGCA R:TCACAGCCCAGAGAGCAGA	NM_214393.1
Q-IFNβ	F:TCGCTCTCCTGATGTGTTTCTC R:AAATTGCTGCTCCTTTGTTGGT	NM_001003923.1
Q-GAPDH	F: TGGAGTCCACTGGTGTCTTCAC R: TTCACGCCCATCACAAACA	NM_001206359.1
Q-IL-6	F: AAATGTCGAGGCCGTGCAGATTAG R: GGGTGGTGGCTTTGTCTGGATTC	NM_001252429

**TABLE 2:** siRNA sequences of targeted genes used in this study

Gene	Sequence (5′–3′)
siPKM2-1	Sense: GGAAAGAACAUCAAGAUAATT Antisense: UUAUCUUGAUGUUCUUUCCTT
siPKM2-2	Sense: GGAAUGAACGUGGCUCGUUTT Antisense: AACGAGCCACGUUCAUUCCTT
siPKM2-3	Sense: GGGUGAACUUGGCCAUGAATT Antisense: UUCAUGGCCAAGUUCACCCTT

### Co-immunoprecipitation and western blot

To investigate the interaction between cellular protein PKM2 and CSFV NS4A/NS5A protein in mammalian cells, the constructed PKM2 with Flag-tagged and EGFP-tagged NS4A/NS5A plasmid were co-transfected into HEK293T cells, PK-15 cells, or 3D4/2 cells. Post-infection was collected 24 h after transfection and washed with cold PBS thrice. The cell membranes were disrupted with IP lysis buffer (Beyotime, P0013) containing 1 mM phenylmethylsulfonyl fluoride (Beyotime, ST506) for 20 min at 4°C prior. Cell lysates were centrifuged at 13,000 × *g* for 10 min at 4°C. The clarified lysate was precleared with protein A + G agarose beads (Santa Cruz Biotechnology, sc-2003) at 4°C for 4 h and then immunoprecipitated with anti-Flag monoclonal antibody (Beyotime, AF5051) for 5 h at 4°C. Then, the immunoprecipitated mixtures were washed four times with PBS (pH 7.4), boiled in sample buffer, and analyzed by western blot with anti-Flag and anti-EGFP antibodies (Beyotime, AG279).

For western blot analysis, tissue samples were collected and lysed on ice for 20 min with 150 µL RIPA cell lysis buffer (Beyotime, P0013B) containing 4 µL protease and phosphatase inhibitor mixture (50×) (Beyotime, P1051). Cell supernatant was discarded when preparing cell samples, and cells were washed three times with PBS. A moderate amount of cell lysis buffer containing proteasome and phosphatase inhibitors (Beyotime, P1045) was added to the cell monolayer and incubated on ice for 15 min. Next, the clarified lysate was centrifuged at 13,000 × *g* 4°C for 20 min. The precipitated protein samples were mixed with 5× protein loading buffer, boiled at 100°C for 10 min, subjected to SDS-PAGE, and transferred to a PVDF membrane (Roche). After blocking in 5% skim milk containing 0.05% Tween 20 (Sigma-Aldrich, 8221841000) at room temperature for 2 h, the membranes were immersed in the prepared primary antibody and then shaken overnight at 4°C. The next day, after washing thrice with phosphate-buffered saline and tween (PBST), the membranes were incubated with appropriate HRP-conjugated goat anti-mouse IgG secondary antibodies (diluted 1:1,000 in PBST) for 1 h at 37°C. Protein bands were detected with the enhanced chemiluminescence Plus kit (Beyotime, P0018 S) by luminescent image (Tanon 6600).

### Confocal immunofluorescence microscopy

To analyze the effects of different treatments or transfections on relevant indicators, the cells were grown in a glass-bottomed 35 mm petri dish (NEST, GBD-35-20). When needed, the indicated plasmid DNA (Mito-mRFP-EGFP) was transfected with p3×Flag-CMV or Flag-PKM2 to analyze the effect of PKM2-induced mitophagy used for transfection. Mitochondria in live cells were stained with 100 mM MitoTracker CMXRos Red (Beyotime, C1049) for 30 min at 37°C.

After proper treatment, the cells were washed thrice with PBS and fixed with 4% paraformaldehyde at 25°C for 30 min with 0.2% Triton X-100 (Sigma-Aldrich, T8787) permeate for 10 min. The cells were blocked in PBS containing 5% bovine serum albumin (Beyotime, ST023) for 1 h at 37℃. Next, the cells were incubated with the indicated primary antibody of rabbit polyclonal antibody (anti-TOM20; 1:500) and a mouse monoclonal antibody (anti-Flag; 1:200) in PBS buffer at 37°C, followed by a 1 h incubation in PBS containing goat anti-mouse and anti-rabbit secondary antibodies (Alexa Fluor 647-conjugated goat anti-mouse secondary antibodies or Alexa Fluor 488-conjugated goat anti-rabbit secondary antibodies) at a dilution of 1:500 at 37°C. Wherever indicated, nuclei were stained with DAPI (Beyotime, C1002). The fluorescence signals were acquired using a TCS SP2 confocal microscope (Leica TCS SP8) with constant excitation, emission, pinhole, and exposure time parameters. Following image acquisition, autophagy was quantified as described by Hariharan et al. (78). In brief, 30 cells were randomly selected, then the number of intracellular autophagosomes (red and green dots, i.e., yellow dots in the merged image) and autolysosomes (dots that are only red but not green, i.e., red dots in the merged image) was counted, and the resultant total count was then normalized against the number of cells, providing the average intracellular dot count. Representative positive cells were marked with a white dotted line and their fluorescence intensity was quantified using ImageJ software. Statistical analysis was performed using GraphPad Prism6 software.

### Quantitative real-time RT-PCR

For targeted gene expression analysis, total RNA was isolated using TRIzol reagent (Invitrogen, 15596026), and cDNA synthesis was performed using the PrimeScript RT reagent Kit (TAKARA, DRR037) according to the manufacturer’s protocol. Then, the cDNA was used as the template for RT-qPCR. Amplification and analyses were carried out using qPCR SYBR Green Master Mix (YEASEN, 11199ES03) in a Bio-Rad CFX96 Real-Time PCR System (Bio-Rad, USA). Relative fold changes in gene expression were normalized against β-actin expression using the 2−ΔΔCt threshold method (79). The primers used are described in Table 1. For CSFV genomic copies’ detection, viral RNA was extracted using the MiniBEST Viral RNA/DNA Extraction Kit version 4.0 (TaKaRa, DV819) and reverse transcribed using PrimeScript RT Master Mix (Perfect Real Time; TaKaRa, RR036A). The resulting cDNA was then amplified using SYBR Premix Ex Taq (Tli RNaseH Plus; TaKaRa, RR420B) and an iQ5 iCycler detection system (Bio-Rad, USA). Each sample was assayed in triplicate.

### Biochemical interventions

3D4/2 and PK-15 cells cultured in 12-well plates were pretreated with DMSO (control), Mdivi-1 (10 µM), or CCCP (10 µM). Then, the cells were cultured in a fresh medium containing the same drug as a pretreatment at different times.

### Pyruvate measurement

Pyruvic acid was measured by ultraviolet spectrophotometry (Solarbio, Beijing, China). The measurement method was carried out according to the instructions of the pyruvate (PA) content determination kit.

### ROS generation test

PK-15 cells were cultured overnight in 6-well plates and transfected with PKM2 for 24 h. DCFH-DA was diluted 1:1,000 with serum-free culture medium to a final concentration of 10 µmol/L. The cell culture medium was removed, an appropriate volume of diluted DCFH-DA was added, and incubated for 20 min at 37°C in a cell culture incubator. The cells were washed thrice with serum-free cell culture medium to remove any DCFH-DA that had not entered the cells. Rosup was added to the positive control wells as a positive control. Usually, the reactive oxygen positive control significantly increased the reactive oxygen levels after stimulating the cells for 20–30 min. The cells were then directly observed using a laser confocal microscope.

### Mitochondrial membrane potential

To measure mitochondrial membrane potential using JC-1 dye, 1 mL of the dye working solution was added to 1 mL of cell culture fluid and the mixture was incubated for 20 min at 37°C. Then, the supernatant was aspirated and the cells were washed twice with JC-1 staining buffer. Two milliliters of the cell culture medium was added, and the cells were observed using a fluorescence or confocal microscope. The images were quantitatively analyzed using Image J software, and statistical analysis was performed using GraphPad Prism software.

### Isolation of mitochondria

PK-15 cells grown to 80% confluence in 10-cm dishes were infected by CSFV and further cultured for 48 h. After washing twice with PBS, the cells were dispersed with trypsin, collected by centrifugation, and the cell suspension was harvested at approximately 850 *g* for 2 min. Mitochondrial isolation of PK-15 cells was performed using reagent-based methods according to the manufacturer’s instructions (ThermoFisher, 89874).

### Cell viability assay

Cell viability was evaluated with the Cell Counting Kit-8 (CCK-8, Beyotime, C0038) according to the manufacturer’s instructions. About 1 × 104 cells (PK-15 or 3D4/2) per well were inoculated in 96-well plates in a CO_2_ incubator at 37°C for 24 h. SiPKM2-1/-2/-3 or SiNC was transfected with Lipofectamine3000 reagent to the cells. After 48 h, cells were cultured in 100 µL fresh medium containing 10 µL CCK-8 solution and incubated at 37℃ for 4 h. Subsequently, The optical density was determined at 450 nm using a microplate reader (Bio-Rad, USA).

### Transmission electron microscopy

To detect the effects of PKM2 on mitochondria, PK-15 and 3D4/2 cells were transfected with PKM2 or control and grown in a 10 cm petri dish. The discarded medium was washed with PBS twice and fixed with an electron microscope fixator at room temperature for 5 min. The cells were then collected in a 1.5 mL microcentrifuge tube with cell curets and centrifuged for 2 min (no more than 3,000 *g*). After discarding the fixator, a new electron microscope fixator was added, and the cell clusters were gently lifted and suspended in the fixator. Cell pellets were dehydrated with an acetone series and embedded in epoxy resin. Next, for observation, the ultrathin sections were prepared and examined by JEM-2010HR transmission electron microscope (JEOL). Five cell samples were randomly selected for each treatment, and the average number of intracellularly contained mitochondrial vesicles was calculated and statistically analyzed using GraphPad Prism6 software.

### Virus titration by immunofluorescence assay

IFAs were performed to estimate the virus titers of CSFV in the cellular supernatant. After the 6-well plate cells grew to 80%, the cells were adequately treated and cultured for 24 or 48 h. Cell supernatant was collected and inoculated into the 96-well culture plate with 10 groups generated by a 10-fold dilution series (10^−1^–10^−10^) for 48 h at 37°C, and eight repetitions for each dilution were performed. A complete culture medium without CSFV was used as a negative control. Cell medium was discarded, and cells were fixed with absolute ethanol at room temperature for 20 min and permeabilized by 0.1% Triton X-100 in a 4°C refrigerator for 20 min. Cells were incubated with an immunofluorescence assay using mouse anti-CSFV E2 antibody (1:200) at 4°C for about 16 h. After washing, cells were incubated with goat anti-mouse IgG–fluorescein isothiocyanate antibody (FITC, 1:500) for 2 h at 37°C. FITC-positive cells were observed and counted under a fluorescence inversion microscope (Nikon). Virus titers were calculated by the Reed–Muench method and are expressed as median tissue culture infective doses (TCID50) per 0.1 mL.

### Animal experiments

The animal infection experiment was performed as described by Zhu et al. (80). In brief, six 2-month-old healthy pigs were randomly divided into two groups of three each. In one group, three healthy 2-month-old pigs were randomly selected and infected with 10^5^ median tissue culture infectious doses (TCID50) of CSFV-Shimen strain. The other three control pigs were injected with the same dose of PBS as the control. Clinical symptoms and rectal temperature of pigs were monitored and recorded daily until most infected animals (10 dpi) developed clinical signs. The animals were euthanized, and samples of heart, liver, spleen, lungs, kidneys, brain, inguinal lymph nodes, mesenteric lymph nodes, thymus, and tonsils were collected for analysis. The expression level of PKM2 was measured by immunohistochemistry.

### Statistical analysis

All results are expressed as the mean ± standard deviation (SD). The intensities of the western blot bands were analyzed with Image-Pro Plus (version 6.0). For statistical analyses, two-tailed Student’s *t*-test was performed for pair-wise comparisons or one-way ANOVA for multiple group comparisons using GraphPad Prism 5 (GraphPad Software). Differences in each group were considered to be significant, with *P* values less than 0.05.

## SUPPLEMENTAL MATERIAL

Supplemental material jvi.01751-23-s0001.docx Fig. S1 and S2; Table S1.

ASM does not own the copyrights to Supplemental Material that may be linked to, or accessed through, an article. The authors have granted ASM a non-exclusive, world-wide license to publish the Supplemental Material files. Please contact the corresponding author directly for reuse.
