# Supplementary material for: PKM2 induces mitophagy through the AMPK-mTOR pathway promoting CSFV proliferation
Source: J Virol. 2024 Feb 6;98(3):e01751-23. doi: 10.1128/jvi.01751-23 (PMC10949426; doi:10.1128/jvi.01751-23)
Supplement: Supplemental material — Fig. S1 and S2; Table S1. [file jvi.01751-23-s0001.docx]

**PKM2 induced mitophagy through AMPK-mTOR pathway promoting CSFV proliferation**

**Xiaodi Liu^1^, Quanhui Yan^1^, Xueyi Liu^1^, Wenkang Wei^2^, Linke Zou^1^, Feifan Zhao^1^, Sen Zeng^1^, Lin Yi^1^, Hongxing Ding^1^, Mingqiu Zhao^1^, Jinding Chen^1^, Shuangqi Fan^1*^**

**1 College of Veterinary Medicine, South China Agricultural University, Guangzhou, Guang Dong Province, China**

**2Agro-Biological Gene Research Center, Guangdong Academy of Agricultural Sciences, State Key Laboratory of Swine and Poultry Breeding Industry，Guangzhou，China**

*** Corresponding author**

Shuangqi Fan

E-mail: [shqfan@scau.edu.cn](mailto:shqfan@scau.edu.cn)

**1 Supplementary Figures**

###
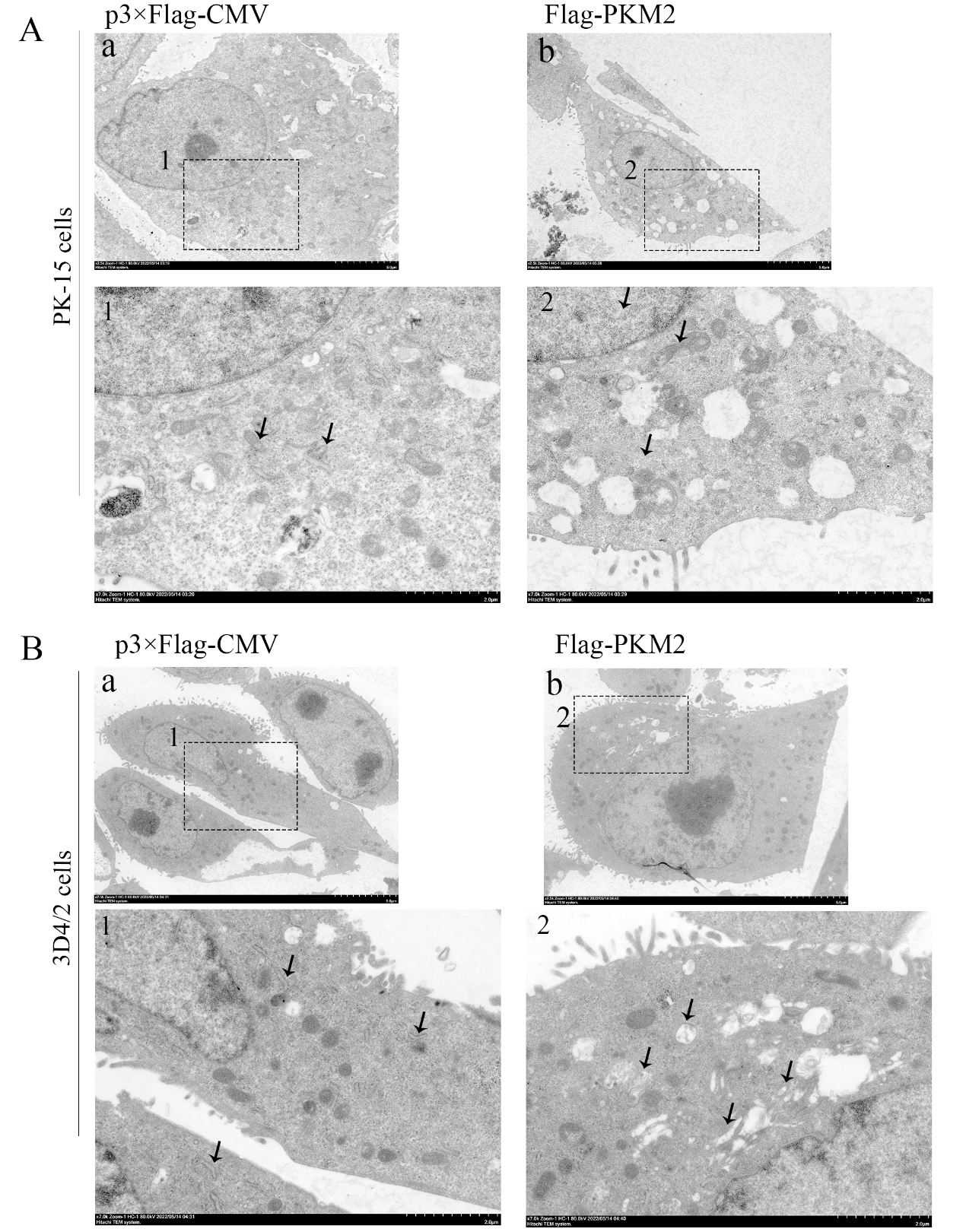


**Supplemental Figure 1.** TEM images revealed the mitochondrial ultrastructure in PKM2-overexpressed cells. PKM2 overexpression led to increased mitochondrial autophagic vesicles in PK-15 (A) and 3D4/2 cells (B). Enlarged images show elongated tubular mitochondria in mock cells and fragmented elliptic mitochondria enclosed by membrane-like vesicles in PKM2-overexpressed cells. Scale bar: 2 μm.

###
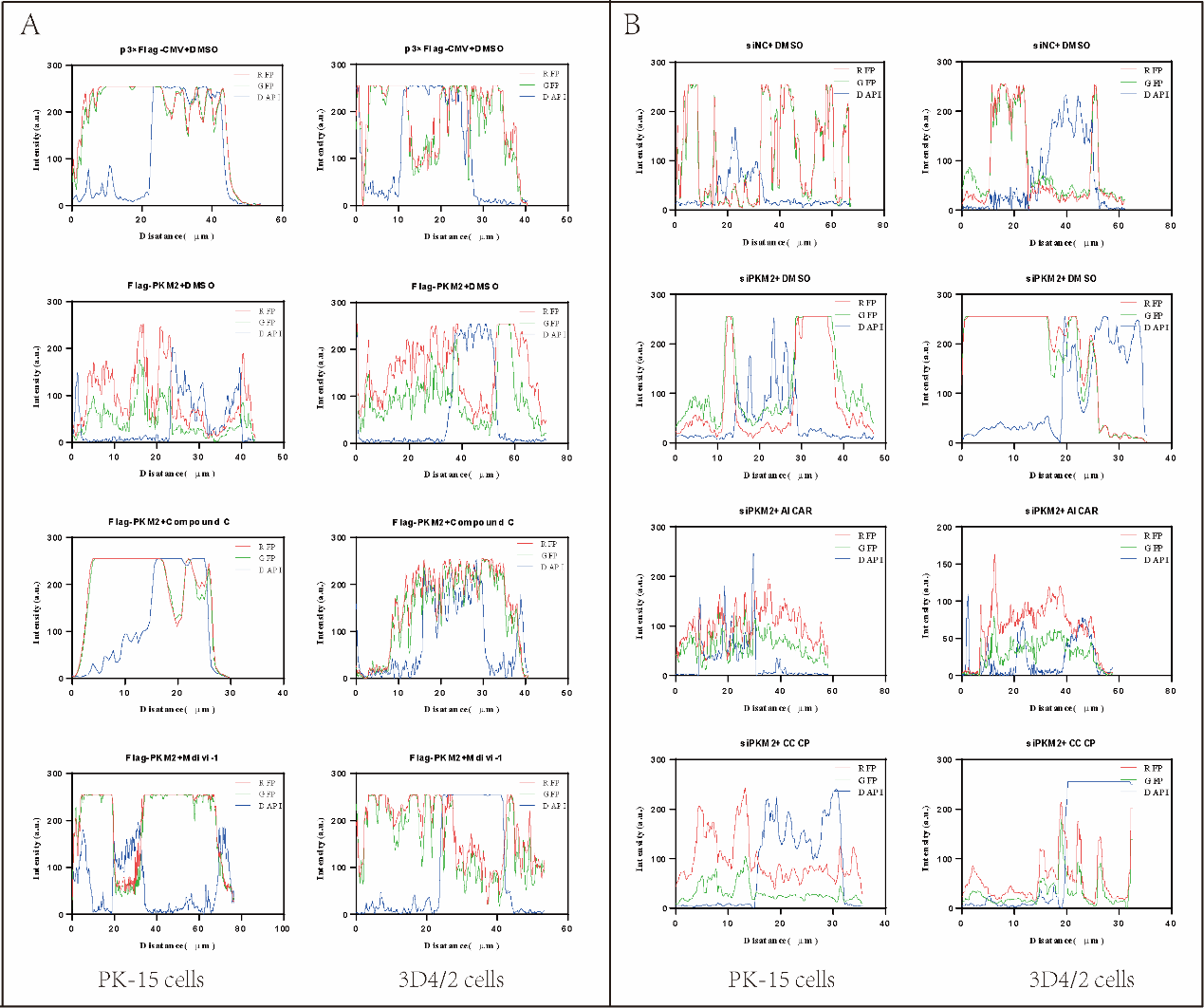


**Supplemental Figure 2.** (C) PK-15 and 3D4/2 cells transiently expressing Mito-mRFP-EGFP were pretreated with DMSO/Compound C/Mdivi-1 (10µM) for 2 h and then transfected with p3×Flag-CMV or Flag-PKM2. (D) PK-15 and 3D4/2 cells transiently expressing Mito-mRFP-EGFP were pretreated with DMSO/AICAR/CCCP (10µM) for 2 h and then transfected with siNC or siPKM2. The fluorescence intensity of ZOOM4 images was quantified using Image-Pro Plus 6.0 software.

**2 Supplementary Tables**

Table S1 NS4A interacting proteins selected by LC-MS/MS in PK-15 cells.

| Number | GenBank accession | Gene symbol | Protein name |
| --- | --- | --- | --- |
| 1 | NM_214303.2 | OAS | 2'-5'-oligoadenylate synthase 1 |
| 2 | XM_001929069.5 | PKM | Pyruvate kinase |
| 3 | NM_213973.2 | HSP90aa1 | Heat shock protein HSP 90-alpha |
| 4 | DQ673096.1 | EEF1A | Elongation factor 1-alpha |
| 5 | NM_001100193.1 | EIF4A3 | Eukaryotic initiation factor 4A-III |
| 6 | XM_021070271.1 | DTX3L | Deltex E3 ubiquitin ligase 3L |
| 7 | XR_306784.3 | UBA7 | Ubiquitin like modifier activating enzyme 7 |
| 8 | XM_005662337.3 | RanBP2 | E3 SUMO-protein ligase RanBP2 |
| 9 | XM_021071510.1 | PFKL | 6-phosphofructokinase |
| 10 | XM_021064068.1 | AKR1CL1 | aldo-keto reductase family 1, member C-like 1 |
| 11 | NM_001114269.1 | ACACA | Acetyl-CoA carboxylase alpha |
| 12 | NM_214319.1 | EIF2AK2 | Eukaryotic translation initiation factor 2 alpha kinase 2 |
| 13 | XM_013989124.2 | ZFP36L1 | ZFP36 ring finger protein like 1 |
| 14 | XM_021066200.1 | LGALS9 | Galectin-9 |
| 15 | NM_001097501.2 | LGALS3 | Galectin-3 |
| 16 | XM_001924813.6 | MCM3 | Minichromosome maintenance complex component 3 |
| 17 | NM_001244512.1 | PCBP2 | Poly(rC) binding protein 2 |
| 18 | XM_021099846.1 | H2B | Recombinant Histone H2B |
| 19 | NM_001113435.1 | PTPN1 | Protein tyrosine phosphatase non-receptor type 1 |
| 20 | XM_005672961.3 | YWHAB | Tryptophan 5-monooxygenase activation protein beta |
| 21 | NM_001244064.1 | DHFR | Dihydrofolate reductase |
| 22 | XM_021066028.1 | ACLY | ATP citrate lyase |
| 23 | NM_001167640.1 | ASNS | Asparagine synthetase |
| 24 | NM_001243210.1 | CANX | Calnexin |
| 25 | XM_021078328.1 | H2AFY | H2A histone family, member Y |
| 26 | NM_214332.1 | RACK1 | Receptor for activated C kinase 1 |
| 27 | XM_005656971.3 | TRIM25 | Tripartite motif containing 25 |
| 28 | NM_213960.1 | VDAC1 | Voltage dependent anion channel 1 |
